# Mapping of six somatic linker histone H1 variants in human breast cancer cells uncovers specific features of H1.2

**DOI:** 10.1093/nar/gku079

**Published:** 2014-01-28

**Authors:** Lluís Millán-Ariño, Abul B. M. M. K. Islam, Andrea Izquierdo-Bouldstridge, Regina Mayor, Jean-Michel Terme, Neus Luque, Mónica Sancho, Núria López-Bigas, Albert Jordan

**Affiliations:** ^1^Department of Molecular Genomics, Institut de Biologia Molecular de Barcelona (IBMB-CSIC), Barcelona, E-08028 Spain, ^2^Research Programme on Biomedical Informatics, Universitat Pompeu Fabra, Barcelona, E-08003 Spain, ^3^Department of Genetic Engineering, Biotechnology, University of Dhaka, Dhaka-1000, Bangladesh, ^4^Centro de Investigación Príncipe Felipe, Valencia, E-46012 Spain and ^5^Institució Catalana de Recerca i Estudis Avançats (ICREA), Barcelona, E-08010 Spain

## Abstract

Seven linker histone H1 variants are present in human somatic cells with distinct prevalence across cell types. Despite being key structural components of chromatin, it is not known whether the different variants have specific roles in the regulation of nuclear processes or are differentially distributed throughout the genome. Using variant-specific antibodies to H1 and hemagglutinin (HA)-tagged recombinant H1 variants expressed in breast cancer cells, we have investigated the distribution of six H1 variants in promoters and genome-wide. H1 is depleted at promoters depending on its transcriptional status and differs between variants. Notably, H1.2 is less abundant than other variants at the transcription start sites of inactive genes, and promoters enriched in H1.2 are different from those enriched in other variants and tend to be repressed. Additionally, H1.2 is enriched at chromosomal domains characterized by low guanine–cytosine (GC) content and is associated with lamina-associated domains. Meanwhile, other variants are associated with higher GC content, CpG islands and gene-rich domains. For instance, H1.0 and H1X are enriched at gene-rich chromosomes, whereas H1.2 is depleted. In short, histone H1 is not uniformly distributed along the genome and there are differences between variants, H1.2 being the one showing the most specific pattern and strongest correlation with low gene expression.

## INTRODUCTION

Eukaryotic DNA is packaged into chromatin through its association with histone proteins. The fundamental repeat unit of chromatin is the nucleosome, which consists of 146 bp of DNA wrapped around an octamer of core histone proteins H2A, H2B, H3 and H4. Linker histone H1 sits at the base of the nucleosome near the entry and exit sites and is involved in the folding and stabilization of the 30-nm chromatin fiber, allowing a higher degree of DNA compaction (1–4). Histone H1 is a family of lysine-rich proteins that consists of three domains: a short basic N-terminal tail, a highly conserved central globular domain and a long positively charged C-terminal tail. Like in core histones, these tails are posttranslationally modified, mainly by phosphorylation, but also by acetylation, methylation, ubiquitination and formylation (5–10). Due to its role in the formation of higher-order chromatin structures, H1 has classically been seen as a structural component related to chromatin compaction and inaccessibility to transcription factors, RNA polymerase and chromatin remodeling enzymes (11,12). However, in recent years, the view that H1 plays a more dynamic and gene-specific role in regulating gene expression is gaining strength. Knock-out or knock-down studies in several organisms have revealed that only a few genes change in expression on complete depletion of H1, some being up- and some downregulated (13–22).

Unlike core histones, the H1 histone family is more evolutionary diverse and many organisms have multiple H1 variants or subtypes, making the study of these proteins more complex. In humans, the histone H1 family includes 11 different H1 variants with 7 somatic subtypes (H1.1 to H1.5, H1.0 and H1X), three testis-specific variants (H1t, H1T2 and HILS1) and one oocyte-specific variant (H1oo). Among the somatic histone H1 variants, H1.1 to H1.5 are expressed in a replication-dependent manner, whereas H1.0 and H1X are replication-independent. H1.2 to H1.5 and H1X are ubiquitously expressed, H1.1 is restricted to certain tissues, and H1.0 accumulates in terminally differentiated cells (23).

It is still far from clear why there are so many H1 variants and great efforts have been made recently to elucidate whether they play specific roles or have redundant functions. Single or double H1 variant knock-out studies in mice did not identify any specific phenotype and this was attributed to the compensatory upregulation of other subtypes, favoring the view that there is redundancy between H1 variants (18). Despite these observations, there is growing evidence supporting the view that histone H1 variants do have specific functions. H1 subtypes present cell type and tissue-specific expression patterns and their expression is regulated over the course of differentiation and development (24–31). Different H1 subtypes have also been differentially related with cancer processes (32–35). Chromatin binding affinity and residence time vary between H1 subtypes owing to differences mainly in the C-t tail, but also in the N-t tail (36–44). Furthermore, H1 subtypes are differently posttranslationally modified and these modifications modulate their interaction with different partners. This could explain some reported specific functions for certain H1 variants (45–57). Finally, global gene expression analyses in various cell types reveal that histone H1 variants control the expression of different subsets of genes, pointing to a specific role of H1 variants in gene regulation (58,59).

To fully understand the function of histone H1 and its variants, several groups have explored the genomic distribution of H1 *in vivo*. Initial biochemical and microscopy-based approaches suggested a nonuniform distribution of H1 in the cell nucleus and found differences between variants (44,60,61). However, due to the lack of specific ChIP-grade antibodies for most of the H1 variants, it has been challenging to identify the precise mapping of H1 variants in the genome. Genome-wide studies with histone H1 started with ChIP-chip experiments in MCF7 cells using an antibody for total H1 (62) and continued using DamID technique for the unique *Drosophila* histone H1 (63). Recently, some groups succeeded in obtaining the first genome maps for H1 variants. The genome-wide distribution of human H1.5 in IMR90 fibroblasts reveals that there are zones of enrichment in genic and intergenic regions of differentiated human cells, but not in embryonic stem cells, associated with gene repression and chromatin compaction (64). Furthermore, analysis of tagged H1c and H1d variants in knock-in mouse embryonic stem cells (ESCs) by ChIP-seq shows depletion of these variants from guanine–cytosine (GC)- and gene-rich regions and active promoters, and positive and negative correlations with H3K9me3 and H3K4me3, respectively, as well as an overrepresentation in major satellites (65). Finally, using DamID technology, the genomic mapping of human H1.1 to H1.5 variants was also achieved in IMR90 cells (66). While H1.2 to H1.5 showed, in general, similar distributions and were depleted from CpG-dense and regulatory regions, H1.1 showed a district profile, pointing to a specific role of this variant in chromatin function.

In this study, we investigated the distribution of the different H1 somatic variants in breast cancer cells by chromatin immunoprecipitation (ChIP) combined with quantitative polymerase chain reaction (qPCR), tiling promoter arrays and high-resolution sequencing. We combined the use of specific antibodies for some variants and hemagglutinin (HA)-tagged recombinant H1 variants expressed in cell lines to study the genome-wide distribution of H1.0, H1.2 to H1.5 and also H1X, a more recently identified and distantly related H1 variant. H1.1 was omitted from our analysis, it being the only somatic H1 variant not present in many cell types, including the cells used here. We also compared H1 distribution with the nucleosome distribution in our T47D human breast cancer cell lines, by H3 immunoprecipitation. Our data support the view that all H1 variants occur across the genome, but also uncover specific features for H1.2, both at promoters and genome-wide. Interestingly, H1.2 enrichment correlates the most closely with gene repression, structural domains of chromatin such as lamina-associated domains (LADs) and regions of low GC content. Overall, the distribution of H1.2 along chromosomes differs from that of other variants including H1.0 and H1X, the two variants most structurally distant within the somatic H1 family. This work represents a comprehensive attempt to investigate for the first time the occurrence and relevance of the different histone H1 variants in the genome of human cancer cells, and provides valuable data to clarify our understanding of the functionalities and heterogeneity of H1.

## MATERIALS AND METHODS

### Cell lines and culturing conditions

Breast cancer T47D-MTVL cells (carrying one stably integrated copy of luciferase reporter gene driven by the MMTV promoter), or derivative cells stably expressing HA-tagged H1 variants (H1-HA), were grown at 37°C with 5% CO_2_ in RPMI 1640 medium, supplemented with 10% FBS, 2 mM l-glutamine, 100 U/ml penicillin and 100 µg/ml streptomycin, as described previously (59). HeLa cell line was grown at 37°C with 5% CO_2_ in Dulbecco's modified Eagle's medium GlutaMax medium containing 10% fetal bovine serum (FBS) and 1% penicillin/streptomycin. MCF7 cell line was grown at 37°C with 5% CO_2_ in Minimum Essential Medium (MEM) medium containing 10% FBS, 1% penicillin/streptomycin, 1% nonessential amino acids, 1% sodium pyruvate and 1% glutamine.

For Phorbol myristate acetate (PMA) experiments, serum-containing Roswell Park Memorial Institute (RPMI) 1640 media was replaced by serum-free media. After 24 h under serum-free conditions, cells were treated with PMA (100 nM) for the indicated time at 37°C.

### Stable expression of HA-Tagged H1 variants

Generation of T47D-MTVL stably expressing HA-Tagged H1 variants was achieved as described previously (59). Briefly, human histone H1 variants were PCR-amplified from genomic DNA and cloned into pCDNA4-HA vector provided by D. Reinberg’s group (NYU Medical School). The complete H1-HA cassette was cloned into the lentiviral expression vector pEV833.GFP provided by E. Verdin (Gladstone Institute) upstream an internal ribosome entry site (IRES)-GFP cassette. Viruses were then produced and cells were infected with pEV833-derived lentivirus. HA-tagged H1 variants-expressing cell lines were selected by sorting in a FACSvantageSE or FACS caliber machine (Becton Dickinson) for green fluorescent protein (GFP)-positive fluorescence.

### ChIP assays

ChIP assays were performed as described previously (67). Briefly, cells were fixed using 1% formaldehyde, harvested and sonicated using a Diagenode Bioruptor to generate chromatin fragments between 200 and 500 bp. To perform the ChIP, 30 µg of chromatin was immunoprecipitated overnight using the indicated antibody. Rabbit IgG (Santa Cruz Biothechnology) was used as a control for nonspecific interaction of DNA. Input was prepared with 10% of the chromatin material used for an immunoprecipitation. Immunocomplexes were recovered using 20 µl of Protein-A magnetic beads from Millipore. Beads with bound antibody/protein/DNA complexes were washed, decross-linked at 65°C overnight and immunoprecipitated DNA was recovered using the IPure Kit from Diagenode.

The following antibodies were used in this study: anti-H1.2 (Abcam 4086), anti-H1X (Abcam 31972), anit-H3 (Abcam 1791) and anti-HA tag (Abcam 9110).

### ChIP-qPCR

Real-time PCR was performed on ChIP and input DNA using EXPRESS SYBR GreenER qPCR SuperMix Universal (Invitrogen) and specific oligonucleotides in a Roche 480 Lightcycler. ChIP values were corrected by the correspondent input chromatin sample. All oligonucleotide sequences used for the amplifications are available on request.

### ChIP-chip assays with Nimblegen promoter array

At least 10 ng of ChIP and input DNA was amplified using GenomePlex Complete Whole Genome Amplification Kit (Sigma) and eluted with GenElute PCR Clean-Up Kit (Sigma). For ChIP-on-chip experiments we used Nimblgen HG18 Refseq Promoter 3x720K array. One microgram of ChIP and input DNA was directly labeled by Klenow random priming with Cy5 and Cy3 nonamers with Nimblegen Dual-color DNA Labeling Kit following manufacturer's user's guide Chip-chip arrays v6.2, and the labeled DNA was precipitated with 1 volume isopropanol. Hybridization mix including 15 µg of labeled DNA was prepared using Nimblegen Hybridization Kit. Arrays were hybridized in Nimblegen Hybridization System 4 Station for 16–18 h at 42°C, and then washed in 1× Wash solution I, II and III. Hybridization buffers and washes were completed using manufacturer's protocols. Arrays were scanned on a Nimblegen MS 200 Scanner per manufacturer's protocol.

ChIP-on-chip raw data was normalized and differential intensity of each probe compared with input control was calculated using the Nimblegen software DEVA. Average fold change (ChIP versus input) each 50 bp bin for a range of −3.2 kb upstream and 800 bp downstream window from RefSeq transcription start sites (TSS) were calculated using in-house Perl script. LOESS smoothed line plot around the TSS were plotted using in-house script written in R statistical programming language. For ChIP-signal heat map, similarly fold change average for each individual RefSeq transcript was calculated and then data were visualized with Java Treeview (68). Functional annotation of target genes based on Gene Ontology was performed using DAVID Software (Database for Annotation, Visualization and Integrated Discovery).

### ChIP-seq

Library preparation for sequencing: ChIP and genomic library preparation was performed using standard Illumina protocols. Libraries were prepared with the ChIP-seq Sample Preparation Kit (Illumina) according to the manufacturer’s instructions. Briefly, 10 ng of ChIP and input DNA were repaired to overhang a 3′-dA and then adapters were ligated to the end of DNA fragments. DNA fragments with proper size (usually 100–300 bp, including adaptor sequence) were selected after PCR amplification, obtaining qualified library for sequencing.

Sequencing, mapping and peak detection: Sequencing was performed with Illumina HiSeq 2000 system. Raw sequence reads containing >10% of ‘N’, or bases with Q ≤ 20 account for >50% of the total were removed and adaptor sequences were trimmed. Identified clean reads were uniquely aligned allowing at best two mismatches to the UCSC (The Genome Sequencing Consortium) reference genome (human hg18) using the program SOAP (version 2.21) (69). Sequences matching exactly more than one place with equal quality were discarded to avoid bias. Read length and read counts of each library are listed in Supplementary Table S1. Peak caller program for histone, SICER (version 1.1) (70), was used with following parameters: redundancy threshold = 1, window size = 200, fragment size = 150, effective genome fraction = 0.75, gap size = 200, false discovery rate (FDR) = 0.01 and Fold Change at least 2. Input subtracted normalized (total mapped library size) WIG files were produced from duplicate removed aligned reads using the program javaGenomicsToolKit.

Binding sites to gene feature annotation: Enriched peaks were annotated to nearest gene (RefSeq genes) using Bioconductor package ChIPpeakAnno (71). Distribution of enriched and depleted regions (peaks) to various genomic features, and continuous ChIP signal profile distribution of reads along the meta-gene were performed using software CEAS (72) and in-house Python and Perl scripts.

Regulatory regions, histone modification peaks, CpG and LADs abundance: Input-subtracted normalized average H1 variants read density in each enriched locations of regulatory regions, histone modification peaks, CpG and LADs were calculated, and representation in box-plot were made using in-house scripts. As a control, a random sample of genomic windows with equal width was used to perform the significance test (Kolmogorov–Smirnov test).

Publicly available genome-wide location data analysis: Public ChIP-seq data, which includes H3K4me1, H3K4me2, H3K4me3, H3K27me3, H3K27ac, H3K9me3, H3K9ac, H3K36me3, P300, CTCF, FAIRE and DNase enriched genomic locations, are taken from ENCODE project. CpG island genomic location information (hg18) and the coordinates of LADs (73) were taken from UCSC database. Publicly available whole-genome data if not available on hg18 version, they were first remapped to the human genome version hg18 using the UCSC coordinate conversion tool (http://genome.ucsc.edu/cgi-bin/hgLiftOver).

Overlap analysis: Overlap of genomic position range data was done using BedTools (74). Overlap means two genomic range data overlap by at least one base.

Average ChIP signal profile: For sequencing data, ChIP signal around center of each given genomic location were calculated by using normalized input subtracted-average tags number in each 50 bp bins in a set window. Relative distance of each tag from above-mentioned position and average signal was determined by using ‘Sitepro’ script of CEAS package (72) and plotting was done in R programming language.

Occupancy of H1 variants at individual chromosomes: Occupancy of H1 variants at all human chromosomes is an average of the input-subtracted ChIP-seq signal in 50 bp windows. Heat map and dendrogram were done with in-house R scripts. Correlation between the occupancy of H1 variants (input-subtracted ChIP-seq signal average of 50 bp genomic windows) and gene expression and gene richness coefficient was done with in-house R scripts. Gene expression for each chromosome was computed as the average of the expression of all the available expressed genes. The gene-richness coefficient (GRC) for each chromosome was calculated as the ratio between the percentage of total genes present in each chromosome and the percentage of base pairs of each chromosome to the total human genome.

### Agilent expression arrays

Total RNA was extracted using High Pure RNA isolation Kit (Roche) according to the manufacturer’s instructions. cDNA was obtained from 100 ng of total RNA using SuperScript VILO cDNA synthesis Kit (Invitrogen). High RNA integrity was assessed by Bioanalyzer nano 6000 assay. For each sample, 100 ng of total were reverse transcribed into cDNA with a T7 promoter and the cDNA was *in vitro* transcribed into cRNA in the presence of Cy3-CTP using the Low input quick Amp kit (Agilent). Labeled samples were purified using RNeasy mini spin columns (Qiagen). Then, 600 ng of cRNA were preblocked and fragmented in Agilent fragmentation buffer and mixed with Agilent GEx Hybridization mix. Hybridization mix was laid onto each sector of subarray gasket slide and sandwiched against an 8 × 65K format oligonucleotide microarray (Human v1 Sureprint G3 Human GE 8x60k Microarray, Agilent design ID 028004) inside a hybridization chamber, which was hybridized overnight at 65°C. Subsequently array chambers were disassembled submerged in Agilent Gene Expression Buffer 1 and washed 1 min in another dish with the same solution with a magnetic stirrer at 200 rpm at room temperature, followed by 1 min in Agilent Gene Expression Buffer 2 with a magnetic stirrer at 200 rpm at 37°C and immediate withdrawal from the solution and air drying. Fluorescent signal was captured into TIF images with an Agilent scanner using recommended settings with Scan Control software (Agilent). Signal intensities were extracted into a tabulated text file using Feature Extraction software (Agilent) using the appropriate array configuration and annotation files. The normalized log2 intensities were obtained using quantile method with normalized expression background correction the Bioconductor Limma package in R.

### Human H1 variants nomenclature

The correspondence of the human H1 variants nomenclature with its gene names is as follows: H1.0, HIF0; H1.1, HIST1H1A; H1.2, HIST1H1C; H1.3, HIST1H1D; H1.4, HIST1H1E; H1.5, HIST1H1B; H1X, HIFX.

## RESULTS

### All H1 variants are nonspecifically present at gene promoters and are depleted from TSS in active genes or on induced gene activation

To determine whether the genomic distribution of human histone H1 differs between variants, we used ChIP combined with semiquantitative PCR (ChIP-qPCR), promoter array hybridization (ChIP-on-chip) and massive sequencing (ChIP-seq). Because there is a limited number of H1-variant–specific ChIP-grade antibodies (only H1.2 and H1X in our hands), we developed T47D-derived cell lines stably expressing HA-tagged versions of each of the five somatic H1 variants expressed in most cell types (H1.0, H1.2, H1.3, H1.4 and H1.5) (see ‘Materials and Methods’ section) (59). These cell lines proliferated similarly to parental cells (data not shown). HA-tagged H1 variants (H1-HA) were expressed at levels lower than or similar to their corresponding endogenous histone, comparably across the different H1 variant-expressing cell lines, and they were incorporated into chromatin (Supplementary Figure S1). In ChIP-qPCR experiments, an anti-HA antibody was used to specifically pull down H1-associated chromatin fragments in cells expressing H1-HAs (Supplementary Figure S2). H1-associated chromatin included gene promoters, coding regions and repetitive DNA, irrespective of which H1-HA variant was immunoprecipitated (Supplementary Figure S3). A few differences were observed between variants, e.g. there were relatively less H1.3 but more H1.4 and H1.5 at alphoid repeats.

**Figure 1.**
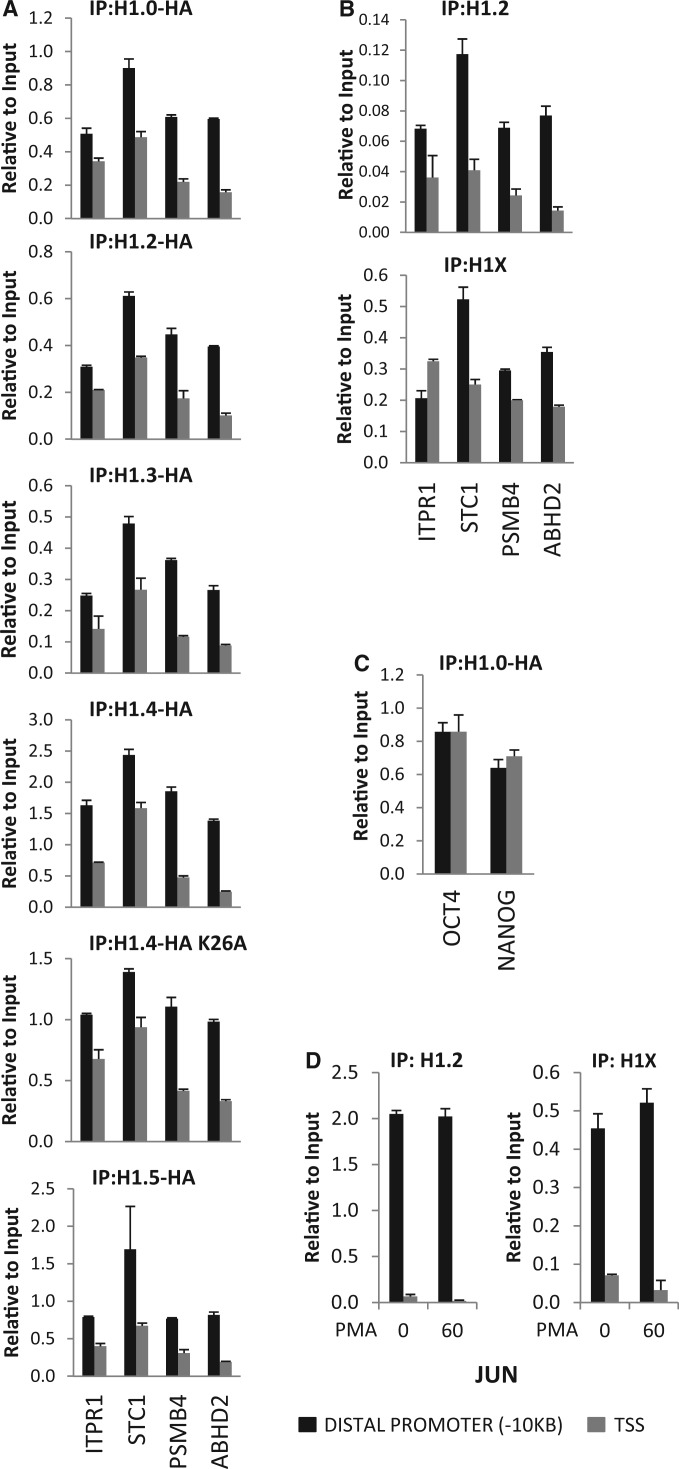
All H1 variants are present at gene promoters and depleted from TSS. (**A** and **C**) ChIP experiments were performed in T47D-derived cells stably expressing HA-tagged H1 variants, wild-type or a K26A mutant of H1.4 with anti-HA antibody and the abundance of IPed material was quantified by qPCR with oligonucleotides for the indicated promoters (−10 kb distal promoter or TSS), and corrected by input DNA amplification with the same primer pair. (**B**) ChIP experiments were performed in parental T47D cells with H1 variant-specific antibodies against H1.2 and H1X and the IPed material was quantified as in (A). (**D**) An H1 valley was performed at TSS of the JUN gene and increased on mitogenic stimulation. T47D cells were treated with PMA 100 nM for 60 min or left untreated and ChIP was performed with H1.2 and H1X antibodies. The abundance of IPed material was quantified by qPCR with oligonucleotides for the JUN promoter (−10 kb distal promoter or TSS), and corrected by input DNA amplification. Representative experiments performed in triplicate are shown.

The specificity of H1 variant distribution was investigated in more detail at gene promoters previously shown to contain H1 in distal regions located 10 kb upstream of their TSS and depletion of H1 at the TSS (H1 valley) (62). All the H1 variants were detected at all distal promoter regions tested, in similar proportions, and a similar degree of H1 depletion was observed at the TSS of all genes for all the H1 variants, including an H1.4 mutant (K26A) at a residue targeted by acetyl and methyl transferases and reported to be involved in recruiting chromatin proteins (Figure 1A) (5,6,46,75). Moreover, local depletion of H1 at TSS was also observed by immunoprecipitating endogenous histones with specific H1.2 and H1X antibodies (Figure 1B). The ChIP specificity of these antibodies was confirmed in H1.2 and H1X inducible knock-down cells (Supplementary Figure S4). Interestingly, the TSS-associated H1 valley was not observed at genes inactive in these cells, i.e. OCT4 and NANOG (Figure 1C), while the H1 valley was evident at genes being expressed, as indicated by mRNA accumulation measured by RT-qPCR. Moreover, the H1 valley correlated with H3K4me3 enrichment at the TSS compared with a 10-kb upstream region, an open chromatin state at TSS measured by formaldehyde-assisted isolation of regulatory elements (FAIRE)-qPCR and nucleosome depletion (H3 ChIP) (Supplementary Figure S5). Furthermore, under stimulating conditions H1 depletion at the TSS was increased at inducible promoters, such as steroid hormone responsive promoters (MMTV) or genes induced by mitogenic agents (JUN and FOS) (Supplementary Figures S6 and S7). Noteworthily, in these early response genes, there was already an H1 valley in noninducing conditions and this became deeper on stimulation (Figure 1D and Supplementary Figure S7).

### Extended depletion of H1 at promoters is dependent on the transcriptional status of the gene and shows differences between variants

**Figure 2.**
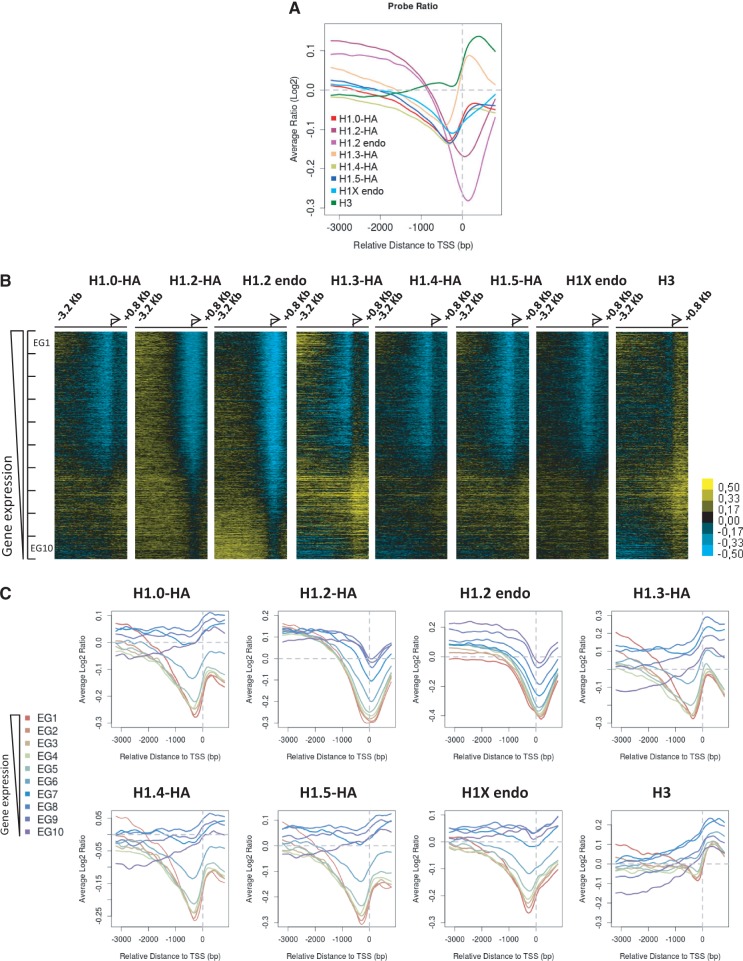
The extension of H1 depletion at promoters is transcription status-dependent and variant-specific. (**A**) Average log2 enrichment ratio of ChIP-chip probe intensity for all transcripts was represented regarding the relative distance to TSS for each variant. (**B**) Heat maps of ChIP-chip probe intensity around TSS (−3200 to +800 bp) for 20 338 transcripts from which the expression rate was determined. Genes are ordered from highest to lowest gene expression. (**C**) Average log2 ratio of ChIP-chip probe intensity represented regarding the relative distance to TSS for all transcripts classified according to expression in 10 groups containing a same number of transcripts, from highest (EG1) to lowest (EG10) expression. Representative ChIP-chip experiments are shown.

To explore the genome-wide distribution of the different H1 variants across gene promoters, we hybridized ChIP material obtained with variant-specific antibodies or corresponding to HA-tagged H1 variant-associated chromatin with a promoter tiling array containing probes for 30 893 transcripts (−3200 to +800 bp to the TSS) arising from 22 542 human promoters. The average log2 ratio of probe intensity for all transcripts was plotted against the relative distance to the TSS for each variant and an H1 valley close to the TSS was apparent in all cases. Interestingly, in the two H1.2 samples (endogenous H1.2 and H1.2-HA), the valley was more pronounced and slightly shifted toward the TSS, compared with that for the other H1 variants (endogenous H1X and H1.0/3/4/5-HA) (Figure 2A).

Subsequently, this ChIP-chip data was combined with gene expression data for ca. 20 000 of the transcripts, obtained with the parental cell line in a human expression array (Agilent) (Supplementary Figure S8), and heat maps representing binding intensity were constructed for each variant, ranking promoters from highest to lowest gene expression (Figure 2B). An H1 valley was clearly seen for at least the top 50–60% most highly expressed transcripts in all variants. Notably, the valley extended toward the least expressed genes in H1.2 samples. Then all the transcripts considered were divided into 10 groups from high to low expression, and average log2 ratio of ChIP-chip probe intensity was plotted against the relative distance to the TSS for each expression group and each variant (Figure 2C). These graphs confirmed that H1 depletion at promoters is dependent on the transcriptional status of the gene. The H1 valley around TSS was deeper and wider for H1.2 than for the other variants, irrespective of whether endogenous or HA-tagged histone was measured. In general, H1 depletion extended to some degree at least 1 kb upstream of the TSS of active genes, further than the predicted extent of the reported nucleosome-free region (NFR) that lies upstream of the TSS. To confirm this result, ChIP-chip for the core histone H3 was also performed and showed that H3 was depleted at active genes and more locally than H1 (Figure 2B and C). H3 and all H1s except H1.2 presented a marked enrichment peak immediately downstream of the TSS, which may correspond to a positioned nucleosome as previously reported (76,77). ChIP-qPCR on selected promoters confirmed some of these observations, namely, in some repressed promoters there was high H1.0 but low H1.2 content around the TSS (Supplementary Figure S9).

In addition to protein-coding genes, the promoter array contained 1145 noncoding transcripts, including structural RNAs and transcribed pseudogenes, that overall presented a low expression rate compared with the complete transcriptome. An H1 valley at the TSS was only apparent on the ChIP-chip heat maps for endogenous and HA-tagged H1.2, in agreement with our observation that an H1.2 valley occurs even at weakly expressed promoters (Supplementary Figure S10).

### H1.2 abundance at distal promoters is a mark of transcriptional inactivity and negatively correlated with the presence of other H1 variants

**Figure 3.**
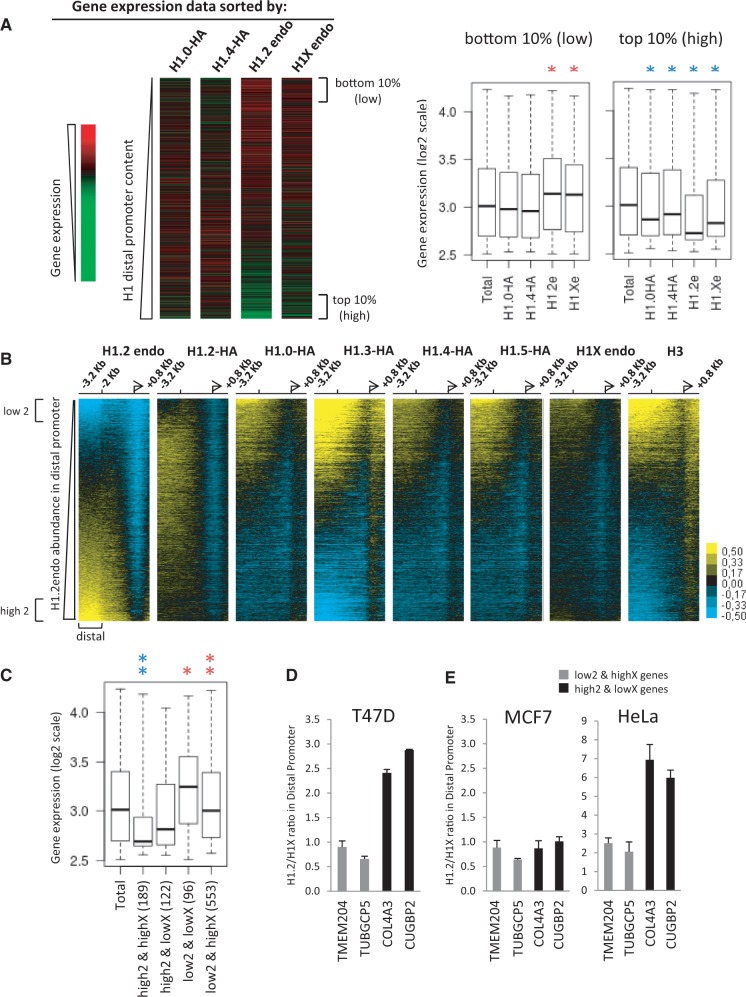
H1.2 abundance at distal promoter regions negatively correlates with gene expression and abundance of other variants. (**A**) Heat maps of gene expression data for 20 338 transcripts ordered from lowest to highest H1 content at distal promoter regions (−3200 to −2000 bp relative to TSS), for each of the H1 variants indicated. (Right panel) Expression levels of genes presenting the highest or lowest H1 variant content at distal promoter is shown as a box plot. Significance was tested using the Kolmogorov–Smirnov test. Enrichment and depletion is marked with red and blue asterisks, respectively. *P < 0.001. (**B**) Heat maps of H1 ChIP-chip probe intensity around TSS (−3200 to +800 bp) for 20 338 transcripts from which the expression rate was determined. Genes are ordered from lowest to highest H1.2 content at distal promoter regions. Genes with the top or lowest distal H1 content are indicated. These genes (2050 genes for each group, 10% of the total) were used to determine the number of coinciding genes as shown in Supplementary Figures S12 and S13. (**C**) Expression levels of coinciding genes in the comparisons between genes presenting the highest or lowest H1.2 or H1X (h2/l2/hX/lX, respectively) content at distal promoter is shown as a box plot. The number of common genes for each comparison is indicated. Significance was tested using the Kolmogorov–Smirnov test. **P < 0.001 and *P < 0.005. (**D**) ChIP-qPCR confirmed that some genes are enriched in H1.2 or H1X at distal promoter. TMEM204 and TUBGCP5 genes were randomly chosen among the group of genes presenting low H1.2 and high H1X (553 genes), and COL4A3 and CUGBP2 genes among the genes presenting high H1.2 and low H1X (189 genes) (see Supplementary Figure S12). After ChIP-qPCR of H1.2 and H1X abundance at distal promoter regions of these genes in T47D cells, the relative ratio H1.2/H1X was calculated. (**E**) The differential ratio between H1.2 and H1X abundance at selected genes observed in T47D cells is conserved in HeLa cells but not in MCF7. Representative ChIP-qPCR experiments performed in triplicate are shown.

Noteworthily, H1.2 abundance at distal promoter regions (−3200 to −2000 bp from TSS) was inversely proportional to gene expression, being more abundant at repressed promoters (Figure 2C). This was also observed to some extent for the other H1 variants and H3 with the exception of the ca. 10% most and least strongly expressed genes that showed the opposite trend. In agreement with this, when gene promoters were ranked from weakest to strongest H1 enrichment at the distal promoter region, a negative correlation with gene expression was seen especially for H1.2 (Figure 3A). Genes with the highest distal promoter H1.2 content (top 10%) mainly fell among those with the lowest expression, whereas genes with the lowest H1.2 content (bottom 10%) fell among those with the highest expression (Figure 3A, right panel). This was partially true also for H1X but less evident for the H1-HAs. Gene ontology analysis of H1 variant-enriched (top 10%) or -deprived (bottom 10%) promoters revealed that different biological processes were regulated by the different variants in T47D cells. For example, genes with the lowest content of H1X at promoters included active genes involved in chromatin organization, and those with the lowest H1.2 content in these regions included genes involved with cell–cell signaling or regionalization. On the other hand, genes with the highest H1X and H1-2 content at promoters included those involved in pattern formation and repressed genes involved in sensory perception, respectively (Supplementary Table S2).

Moreover, H1.2 abundance at distal promoter regions was inversely correlated with H3, H1X and H1-HA abundance, while H1.2-HA showed an intermediate pattern (Figure 3B and Supplementary Figure S11). This indicates that there is a preferential binding of H1.2 in some promoters (mostly repressed genes) compared with the other variants, and vice versa, many promoters are devoid of H1.2 but contain other H1 variants.

Venn diagrams were drawn for the top 10% genes with high or low H1.2 and high or low H1X at the distal promoter to identify genes presenting high2/lowX and vice versa (Supplementary Figure S12). The largest overlaps were between low2/highX promoters (553 genes), mainly corresponding to expressed genes (Figure 3C). Representative genes of the two groups were randomly selected (TMEM204 and TUBGCP5 for low2/highX, and COL4A3 and CUGBP2 for high2/lowX-containing promoters) and used to confirm by ChIP-qPCR that some promoters preferentially bind with particular variants (Figure 3D). Similarly, Venn diagram comparisons of the top 10% genes with high or low H1.2 versus high or low H1.0-HA showed that the largest overlaps were low2/high0 with 716, and high2/low0 with 276 genes (Supplementary Figure S13). Taken together, our data indicated that promoters having few H1.2 variants are loaded with large amounts of other variants, not only with exogenously expressed H1.0-HA but also endogenous H1X. Expression analysis of such groups of genes found that genes with few H1 variants at distal promoters are highly expressed, and vice versa, but also that H1.2 content is the strongest predictor of gene expression (Figures 3C and Supplementary Figures S12C and S13C).

The universality of the relative H1.2/H1X abundance at representative genes was tested in two additional cell lines by ChIP-qPCR (Figure 3E and Supplementary Figure S14). HeLa cells showed results similar to T47D, i.e. H1.2/H1X ratios were higher in COL4A3 and CUGBP2 genes than TMEM204 and TUBGCP5, although ratios in all genes were higher than in T47D reflecting a higher relative abundance of H1.2 in HeLa cells (Supplementary Figure S14). On the other hand, H1.2/H1X ratios in MCF7 were similar in all four genes, due to higher H1X signals in COL4A3 and CUGBP2 genes. This result indicated that relative abundances between variants at promoters were not fully conserved between cell types, although the patterns in T47D and HeLa were similar. In relation to this, ChIP-chip of H1.2 and H1X in HeLa confirmed that these two variants do not coexist at exactly the same distal promoters (Supplementary Figure S15).

**Figure 4.**
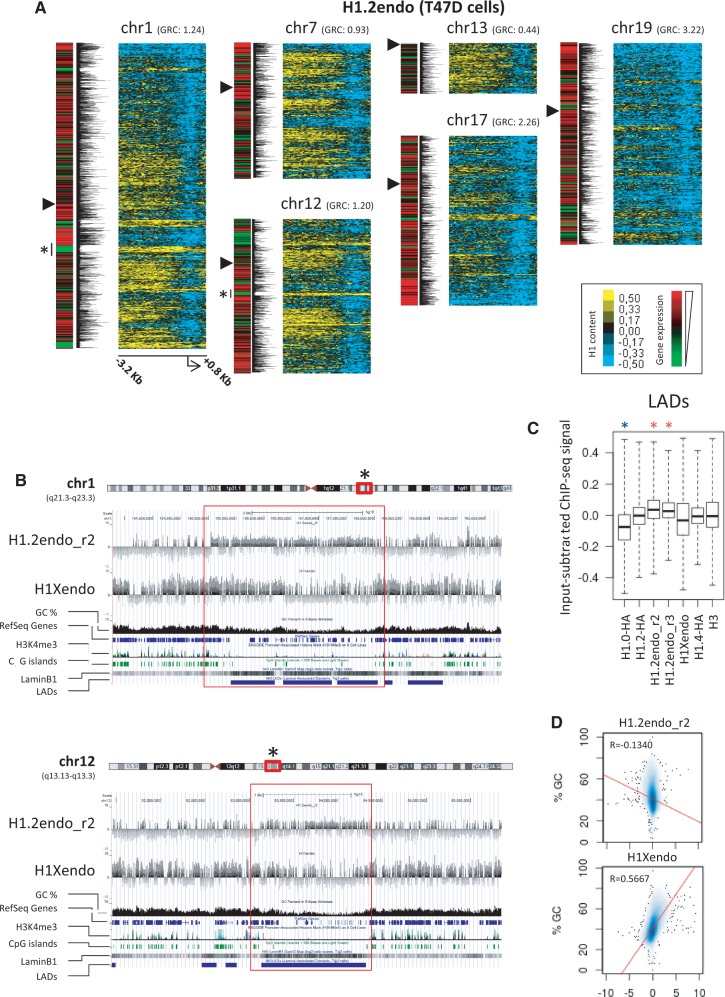
H1 variant content at gene promoters along human chromosomes and relation of H1 variants with LADs and GC content. (**A**) Heat maps of H1.2 ChIP-chip probe intensity around TSS (−3200 to +800 bp) for genes ordered according to their position along several human chromosomes. Gene expression levels for each gene in T47D cells is represented in the left in two different ways (as a heat map and graphical representation of log 2 ratios). A GRC for each chromosome, calculated as the ratio between the percentage of genes present in each chromosome and the percentage of base pairs of each chromosome to the total human genome, is indicated. The centromere location is marked with a triangle. Regions of interest are marked with an asterisk and viewed in the UCSC genome browser in (**B**). (B) Distribution of H1 variants along selected regions of chromosome 1 and 12. Input-subtracted H1.2 and H1X ChIP-seq signal viewed in the UCSC genome browser together with GC content, RefSeq genes, H3K4me3 (ENCODE average of 9 cell lines), CpG and LADs (data from Tig3 lung fibroblasts). (**C**) Box plots showing the occupancy of H1 variants (input-subtracted ChIP-seq signal) within LADs. Significance was tested using the Kolmogorov–Smirnov test taking as a control a random sample of windows with equal width to the LADs. Enrichment and depletion is marked with red and blue asterisks, respectively. *P < 0.001. (**D**) Genome-wide correlation scatterplots of H1.2 and H1X variants versus GC content. X axes: average input-subtracted H1 signal (normalized to 1000 bp window). Y axes: GC%. R: Pearson's correlation coefficient.

Next, we plotted heat maps of H1.2 abundance at the promoters of genes ranked according to their position along several human chromosomes (Figure 4A). Interestingly, several domains of high H1.2 abundance were detected along these chromosomes, correlating with clusters of differential gene expression. Notably, chromosome 19, the most gene-rich chromosome, showed overall high gene expression and low H1.2 content at promoters, as did chromosome 17. On the other hand, the least gene-rich chromosome, chromosome 13, presented low gene expression and high H1.2 content (Figure 4A and Supplementary Figure S16). The observed clustered distribution was well conserved between cell lines, but differed between H1 variants. H1X and H1.0-HA abundances were not clustered with the same pattern as gene expression. Rather, these variants were abundant at promoters located on gene-rich chromosomes 17 and 19, and depleted on the gene-poor chromosome 13 (Supplementary Figure S16). In summary, H1.2 content at promoters is the best H1 reporter of gene expression.

### H1 variants are differentially depleted from regulatory regions and enriched at CpG sites

**Figure 5.**
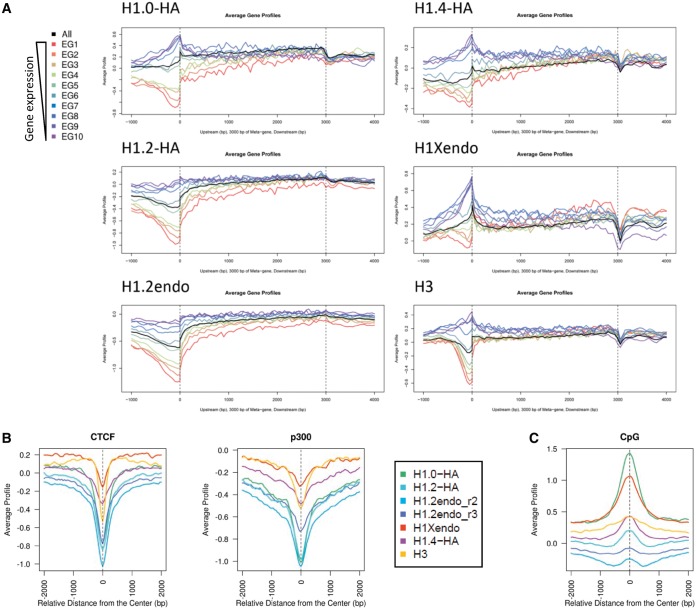
H1 is depleted from regulatory regions but present at CpG sites in a variant-specific manner. (**A**) Average, input-subtracted ChIP-seq signal of H1 variants around gene bodies flanked by TSS and transcription termination site (TTS), grouped according to basal expression (10% of total genes in each group). EG1 represents top expressed genes and EG10 genes with the lowest expression. Average for all genes is shown in black. Genic regions are represented as a 3-kb-long meta-gene surrounded by 1 kb region upstream TSS and 1 kb downstream TTS. (**B**) Average, input-subtracted ChIP-seq signal of H1 variant around the center of genomic CTCF and p300 binding sites (data from T47D cells). (**C**) Average, input-subtracted ChIP-seq signal of H1 variant around the center of CpG islands (as defined in UCSC database).

To further explore whether the genomic distribution of H1 variants is heterogeneous, we combined ChIP of endogenous H1.2, H1X, H3 and HA-tagged H1.0, H1.2 and H1.4 with high-resolution sequencing (ChIP-seq) of up to 50 million reads per sample (Supplementary Table S1). To confirm the results obtained by ChIP-chip, we focused first on the input-subtracted normalized average ChIP signal obtained around coding regions of genes grouped according to basal expression as before (Figure 5A). Again, the H1 valley at the TSS depended on expression rates and differences were seen between H1 variants, mainly the abundance of H1.2 at the TSS of nonexpressed genes being lower than that of the other subtypes, which showed high levels toward nucleosome +1. Transcription termination sites (TTS) also showed differences between variants, being depleted of H1 subtypes except for H1.2. Interestingly, the H1 content of gene bodies increased toward the end and also depended on gene expression rates. While H3 levels were uniform, those of H1 variants such as H1.2 were lower at the 5′ moiety of highly active genes (Figure 5A).

In addition to the local displacement of H1 from active promoters, H1 variants were markedly depleted from other regulatory regions along the genome, namely, CCCTC-binding factor (CTCF) binding sites corresponding to insulators, and p300 binding sites associated with enhancers, but little affected at DNase hypersensitivity sites and FAIRE-identified regions representing open chromatin (Figure 5B and Supplementary Figure S17). When we calculated the input-subtracted coverage of H1 variants across the peaks of selected core histone modifications, depletion of H1.0 and H1.2, and to some extent of H1.4 but not H1X, was associated with positive histone marks linked to strong enhancers such as H3K4me1, H3K4me2 and H3K27ac (Supplementary Figure S17). H1 abundance at H3K4me3 and H3K9ac sites, enriched at TSS of active promoters, differed between variants, reflecting H1.2 depletion at the TSS of most genes but local enrichment of the other variants immediately after the TSS. No strong enrichment of H1 was found at negative histone marks such as H3K9me3 or H3K27me3. It is also worth noting that H1.2 abundance was lower at active marks than at those related with repression and chromatin compaction, in agreement with the observed correlation between H1.2 content and gene repression.

Next, we investigated whether the location of H1 variants coincided with CpG regions across the genome. As seen in Figure 5C, H1.0, H1X and H1.4 were clearly overrepresented in CpG regions compared with H1.2. Because CpG are mostly localized at gene promoters, this finding may reflect the overall higher abundance of those variants compared with H1.2 around TSS, considering the weakly expressed genes. Alternatively, it is not possible to rule out a certain relationship between H1.0 (and other variants apart from H1.2) and CpG or DNA methylation.

### Differential prevalence of H1 variants along the genome

To further correlate ChIP-chip data of H1 abundance at promoters with ChIP-seq signals, regions of clustered genes with high H1.2 content such as the ones marked with asterisks in Figure 4A (chromosomes 1 and 12) were explored for input-subtracted H1 variant content using the UCSC genome browser (Figure 4B). The whole domain was enriched in H1.2 ChIP-seq signal compared with neighboring regions, indicating that H1.2 enrichment was not limited to the promoters of repressed genes therein. Interestingly, this domain was characterized by low GC content and the presence of LADs reported to anchor chromatin segments to the nuclear periphery (73). LADs are typified by low gene-expression levels, representing a repressive chromatin environment. Notably, the distribution of the other variants analyzed by ChIP-seq was not as clearly delimited to this domain as H1.2. Further examination of H1 variant signals across several regions containing LADs using the UCSC genome browser showed that H1.2 was the variant most strongly correlated with LAD positions and had fairly well delimited borders of enrichment (Supplementary Figure S18). When the input-subtracted coverage of H1 variants across LADs was calculated, H1.2 was the only variant showing enrichment (Figure 4C).

We then examined individual chromosomes for the presence of the input-subtracted signal of the different H1 variants. Abundance of H1 was heterogeneous along chromosomes, showing extensive patches of enrichment or depletion of H1 compared with the input (Supplementary Figure S19). Interestingly, the H1.2 pattern was the most different from the other variants, endogenous H1X and HA-tagged variants showing patterns that were similar to each other and to that of H3, while the pattern for HA-tagged H1.2 was more similar to endogenous H1.2 than to other HA-tagged H1s. It is worth noting that long genome patches of low GC content were found to be devoid of all H1 variants except H1.2, which was enriched. We next performed genome-wide correlation analysis between the input-subtracted H1 variant signal and GC content. Low CG content was associated with high occupancy of H1.2 but low occupancy of the other variants, including H1X, and vice versa (Figure 4D and Supplementary Figure S20).

**Figure 6.**
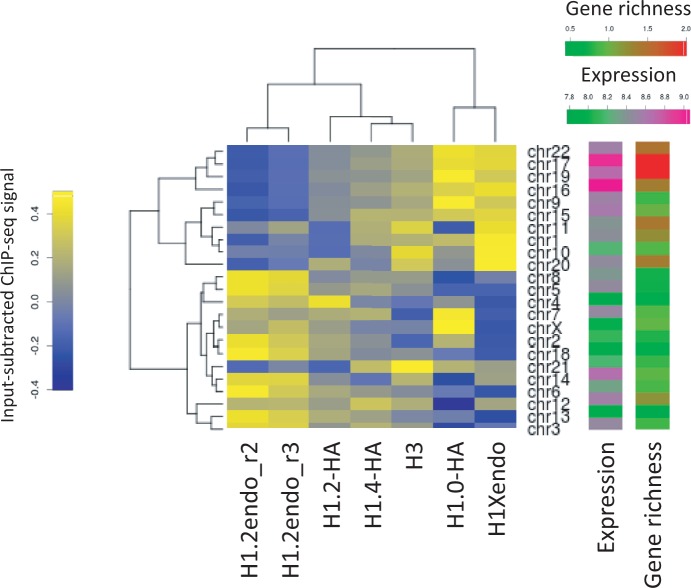
Human chromosomes show enrichment of different H1 variants in relation to average gene expression and gene richness. Heat map and dendrogram of the occupancy of H1 variants (input-subtracted ChIP-seq signal average of 50 bp genomic windows) at individual chromosomes. Gene expression and gene richness coefficient (GRC) of all chromosomes are shown as heat maps. GRC ≥ 2 are shown in the same color.

Further comparison of the overall abundance of H1 variants at each individual chromosome revealed unique patterns creating corresponding clusters of chromosomes and H1 variants (Figure 6). Interestingly, chromosomes were clustered in a manner that was related to their gene-richness and the overall expression of genes they contained. Gene-rich chromosomes showed H1.0 and H1X enrichment, and H1.2 depletion, whereas the opposite was found at gene-poor chromosomes, in agreement with the promoter ChIP-chip data described above. Correlation analysis confirmed these conclusions (Supplementary Figure S21). Notably, H1 variants were clustered differently depending on whether they were replication-independent (H1.0 and H1X) or synthesized over the course of DNA replication only (H1.2, H1.4 and the core H3 histone). Further, H1.0 and H1X had a more heterogeneous distribution between chromosomes (data not shown).

### Genomic annotation of enriched or depleted regions of individual H1 variants shows that H1.2 is associated with intergenic regions and repressed genes

Next, we searched specific regions of the genome either enriched or depleted for each H1 variant signal over input DNA with a fold change ≥2 using SICER software (Supplementary Table S3). Most H1-enriched regions were inside genes (arbitrarily defined as from −5 kb upstream to +3 kb downstream of the TTS), whereas H1.2 peaks were more abundant at intergenic regions (Supplementary Table S3 and Supplementary Figure S22). On the other hand, all H1-depleted regions were more abundant inside genes, especially for H1.2. Within genes, H1.2-enriched regions were disfavored at promoters (−5 kb to +1 kb flanking TSS) compared with other H1 peaks, whereas H1.2-depleted regions were strongly favored, in agreement with ChIP-chip data presented in Figure 2. In agreement with our aforementioned data, the GC content in H1.2-enriched regions was lower than in the other variants (Supplementary Figure S20). Next, we analyzed the overlap between H1-enriched and depleted regions with CpG islands. CpG islands were enriched at H1.2-depleted regions and at regions enriched for the other variants, confirming the inverse correlation between CpG islands and H1.2 described above (Supplementary Figures S22 and S23). As expected, regions overlapping with CpG sites were preferentially located at promoters. For example, 42% of H1.0- or H1X-enriched regions located at promoters overlapped with a CpG island, while this was the case for only 4–8% of regions enriched in these variants located at intergenic regions.

**Figure 7.**
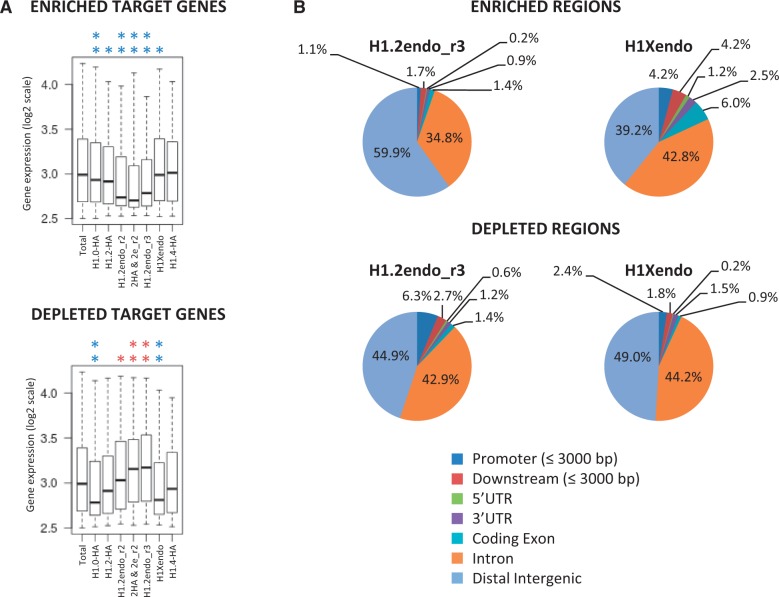
Genomic annotation of regions found to be enriched or depleted of individual H1 variants and expression of target genes. (**A**) The expression profiles of target genes containing enriched or depleted regions for a unique variant are shown as box plots. The profile of genes containing both H1.2-HA and H1.2endo (replica 2) enriched or depleted regions are also shown (2HA & 2e_r2). Significance was tested using the Kolmogorov–Smirnov test. Enrichment and depletion is marked with red and blue asterisks, respectively. **P < 0.001 and *P < 0.005. (**B**) Genomic annotation of regions enriched or depleted of endogenous H1.2 or H1X. Pie diagram of distribution of H1 variants enriched regions at genes, proximal regulatory regions and distal intergenic regions. Promoter and downstream regions are defined as 3000 bp upstream TSS or downstream TTS, respectively. The proportions of the H1.2 and H1X enriched or depleted regions in several genomic features were significantly different from the whole genome proportions of those features (*P* < 2.2e-16). Significance was tested using in-house R scripts.

To identify H1 variant target genes we looked for genes that had at least one H1-enriched region from −5 kb to +3 kb from the TTS. H1.2 was the variant that was found to have the smallest number of target genes (Supplementary Table S3). Overlap analysis disclosed the number of genes containing peaks of a single variant or several variants (Supplementary Figure S24), and expression analysis revealed that genes with only H1.2 peaks were less expressed than target genes containing peaks of any other H1 variant (Figure 7A), in agreement with data above showing lower expression of genes containing elevated levels of H1.2 at distal promoter or coding regions. In those genes, the peak tended to be outside the promoter for H1.2, but at the promoter for the other single variant target genes (Supplementary Table S3). On the other hand, genes presenting H1.2-depleted regions were highly expressed, while genes with depleted regions of H1.0, H1.4 or H1X were expressed at lower levels than the total transcriptome average (Figure 7A).

We further investigated whether the identified H1-enriched regions fell within genes, proximal regulatory regions or distal intergenic regions using CEAS software (70). Again, H1.2 was more differently distributed than the other variants analyzed. H1.0-HA, H1X and H1.4-HA peaks were overrepresented in promoters, UTRs, exons and downstream regulatory regions, and underrepresented in distal intergenic regions compared with the complete genome, whereas H1.2-enriched regions were overrepresented in intergenic regions and underrepresented in exons and promoters (Figure 7B and Supplementary Figure S25). Except for those for H1.2, H1 peaks were as abundant in introns as in distal intergenic regions. On the other hand, depleted regions were similarly distributed across compartments in the different H1 variants, except H1.2-depleted regions, which were more abundant at promoters and less so at intergenic regions.

In summary, our data shows that histone H1 is not uniformly distributed along the genome and there are differences between variants, H1.2 being the one showing the most specific pattern and strongest correlation with low gene expression in breast cancer cells.

## DISCUSSION

### Mapping of H1 variants by ChIP with variant-specific antibodies and protein tagging uncovers differences between H1.2 and the other variants in breast cancer cells

Herein, we have investigated the distribution of all somatic histone H1 variants present in breast cancer cells, i.e. H1.0, H1X and H1.2 to H1.5 by combining ChIP with genomic technologies such as tiling promoter array hybridization and high-resolution sequencing. After testing several H1 variant-specific antibodies that we and others have produced, only H1.2 and H1X commercial antibodies were found to be useful in the ChIP-qPCR experiments, and variant specific, as shown by performing ChIP experiments in H1.2 and H1X knockdown (KD) cells. Consequently, we generated stable cell lines expressing HA-tagged versions of the H1 variants at protein levels close to or below endogenous levels, despite mRNA levels of exogenous H1 forms being higher (data not shown). This suggests that H1 is tightly posttranscriptionally regulated to control the overall levels of H1 and the proportion between variants, which vary considerably across cell types and cell lines. HA-tagging allowed us to perform ChIP of all variants with the same antibody, ruling out the variability being due to diverse antibody specificity or affinity. We found that all the H1 variants studied are widely distributed along the genome and within promoters with few differences between HA-tagged H1.0, H1.3, H1.4 and H1.5. In contrast, endogenous H1.2 presents striking differences. We rule out the possibility that the differential distribution is due to antibody usage or protein overexpression, as endogenous H1X presented an occurrence similar to HA-tagged variants and exogenous H1.2-HA resembled its endogenous counterpart more closely than the other H1-HAs.

On this basis, we report that, in the cell line investigated, H1.2 presents a variant-specific distribution and may have differential functions. In fact, we reported elsewhere that H1.2 KD produces unique effects, namely, cell cycle arrest at G1 and decreased nucleosome spacing, not seen in other H1 KDs, and these were observed not only in T47D cells but also in MCF7 cells (59). Nonetheless, this feature was not general, as it was not seen in other cell types tested, including HeLa cells in which H1.2 is highly abundant, indicating that H1 variants may have cell type-dependent specific effects. Instead, our data cannot rule out that the other variants studied may have redundant functions and distribution in breast cancer cells. A recent report on the genomic distribution of Dam-H1.1 to H1.5 in lung fibroblasts IMR90 cells found that H1.1 is the only subtype showing divergent features (66). H1.1 is not expressed in breast cancer cells or in many other cell types. Instead, H1.2 and H1.4 are the only variants that have been found in all cell lines tested to date (29,78). Additionally, mRNA levels of these two variants are maintained in nondividing cells and along differentiation, compared with H1.3 and H1.5 levels that are reduced (31,79). Although too small a sample, these results suggest that different H1 subtypes may play different roles in different cell types, over the course of development and in cancer cells, inviting further investigation of H1 variants occurrence.

We have noticed that H1.2-HA was not distributed in exactly the same way as endogenous H1.2 and showed intermediate features somewhat similar to the other H1-HAs. We believe that this recombinant protein has the H1.2 structural features that direct it to the natural H1.2-occupied sites, but owing to its overexpression it may also locate at distinct sites normally occupied by other H1 variants. We have observed, by ChIP, that on knock down of endogenous H1.2, H1.2-HA occupancy increased (data not shown), suggesting a relocation to H1.2 sites. Overall, we believe that caution should be taken when interpreting data generated with exogenous histone variants fused either to the Dam domain or to peptide tags.

### H1 depletion from promoters and coding regions is more pronounced than H3 depletion and shows differences between H1 variants

Our analysis has also shown that all H1s are removed from active promoters, with maximum depletion close to TSS but extending several nucleosomes upstream, beyond the reported NFR, and within the coding regions. These regions containing nucleosomes but not H1 may coincide with H2A.Z and H3.3-containing nucleosomes, as both H2A.Z and H3.3 have been reported to locate at active promoters surrounding the NFR, where they positively regulate transcription (80–82). Additionally, other authors have observed weaker histone H1 binding in H2A.Z-containing nucleosomes (83) and a negative genome-wide correlation between H1 and H3.3 (63). These observations support the view that H1 removal is part of the chromatin remodeling events that occur on promoter activation to facilitate binding of transcription factors and the RNA polymerase machinery (49,84–86). Furthermore, the shape of the H1.2 (and H1.2-HA) valley at the TSS in ChIP-chip and ChIP-seq data (Figures 2 and 5) was slightly different from that of other H1 variants. Unlike the signals for other variants, the H1.2 signal did not show local enrichment immediately after the TSS. This local enrichment may coincide with a well-positioned nucleosome (+1), flanked by phased nucleosomes. This indicates that such a nucleosome may contain any H1 variant except H1.2. Additionally, H1.2 was not abundant around the TSS of repressed genes, suggesting that TSS of genes are epigenetically marked, including the absence of H1.2. Overall, we have shown a strong rejection of H1.2 from the TSS of most genes.

Interestingly, we have found that immediate-early responsive promoters, under nonstimulating conditions, are prepared to respond to stimuli by keeping the TSS free of H1, indicating that mechanisms other than transcription initiation might dictate H1 clearance. In this case, there is also histone H3 depletion at the TSS compared with at the distal promoter in the absence of stimuli, indicating that the NFR might be maintained to allow rapid response after stimulation. Supporting our hypothesis, it has been recently proposed that transcription factors interact with DNA in a dynamic way, and some transcription factor–DNA interactions are established before the stimuli, especially at immediate-early genes (87).

Comparison of H1 occupancy with H3 has shown that all H1s except H1.2 follow the distribution of the core histone, whether this represents nucleosome enrichment, stability or defined positioning through the cell population. Nonetheless, H1 depletion at promoters and regulatory sites (CTCF or p300 binding sites) is more extensive than H3, denoting that nucleosomes might be ejected from delimited sites such as the NFR at the TSS, but H1 might be depleted from larger regions encompassing several nucleosomes. This is in agreement with previous reports showing that dips of low H1 occupancy at TSS and regulatory sites are not due to a lack of nucleosomes as they show enrichment of the core histone variant H3.3 (63). Moreover, at coding regions, the differential content of H1 in active versus repressed genes is more pronounced than those of H3, especially toward the 5′ of genes. Consequently, gene-rich domains might adopt an overall decondensed chromatin structure. Nonetheless, at active genes H1 is less abundant in promoters than coding regions, indicating that H1 presence might be more restrictive for transcription initiation than for elongation.

Initial ChIP-qPCR experiments indicated that all H1 variants were present at all tested promoters. Nonetheless, hybridization of ChIP material with a promoter array revealed that promoters might present differential H1 variant abundance (Figure 3). The most striking difference is between H1.2 and the other H1s, including H1X. Subsets of genes with the highest abundance of one variant and the lowest of another have been identified, i.e. those with a high or low H1.2/H1X ratio. Overall, expression of genes presenting these features is different, relating H1 variant content with gene expression. Notably, the relative abundance of H1.2 and H1X in the selected promoters was conserved in the distant HeLa cell line, but not in MCF7 cells. Thus, we propose that the relative promoter abundance of H1 variants may be related to, among other factors, their relative H1 variant content in a given cell type.

### Two types of H1-containing chromatin are present in breast cancer cells with different association with gene density and expression

The negative correlation observed between gene activity and H1.2 content found at promoters extended upstream toward the whole genomic region. Patches of H1.2 enrichment seem to be associated with gene repression, gene-poor regions (including entire chromosomes, such as chromosome 13), low GC content or LADs, features related to chromatin compaction (Figure 4). Moreover, H1.2-enriched regions were frequently found at intergenic regions. Similar results were found in previous studies, linking histone H1 to repressive and compacted regions of the genome and suggesting a role for H1 in 3D organization of the genome. Some of these features were described by Cao et al. for mouse H1c^Myc^ and H1d^FLAG^ in ESCs, the closest orthologs of human H1.2 and H1.3, by Li et al. for human H1.5 in differentiated IMR90 fibroblasts, and by Izzo et al. for human Dam-H1.2 to H1.5 in IMR90 cells also (64–66). However, in the last of these, H1.1 presented a DamID binding profile distinct from the other subtypes that, in some extent, resembles the distribution of H1 other than H1.2 in our analysis in breast cancer cells, that is, they were more closely associated with higher GC content, genes, its promoters and CpG islands, and were not enriched in LADs. Interestingly, in the study of Cao *et al.* when single peaks for H1c and H1d in mouse ESCs were compared, H1d (H1.3) was more closely related to GC-rich sequences and LINES, and H1c (H1.2) to AT-rich sequences, Giemsa-positive regions and satellite DNA. It is conceivable that there are at least two groups of H1 variants with different distributions in each cell type, such that taken together histone H1 variants cover the whole genome, being present in most of the nucleosomes.

Whether a single variant may present distinct features in different cell types rather than having intrinsic properties is an intriguing question. Factors involved may be the relative and absolute abundance of each variant and whether a genome needs more plasticity or is progressively silenced, i.e. pluripotency versus terminal differentiation. In this sense, Li et al. described the existence of zones of H1.5 enrichment in differentiated fibroblasts but not in ESCs (64), and it has been reported that architectural proteins, such as HP1 and H1, are hyperdynamic and bind loosely to chromatin in ESCs (88,89). Additionally, we have previously reported progressive changes in the expression and abundance of H1 variants over the course of differentiation of human embryonic stem cells and of reprogramming of differentiated cells to Induced pluripotent stem cells (iPS), i.e. the opposite direction (31). Thus, considering the importance of H1 in chromatin structure and compaction, differential expression and/or distribution of H1 variants could mediate the transition between different chromatin states, and explain the more ‘open’ chromatin state of undifferentiated cells, which contributes to the maintenance of pluripotency by creating a poised chromatin state that leads to rapid activation of lineage-specific genes when differentiation is induced. In fact, it has been proposed that different ‘anti-silencing’ mechanisms, including incorporation of specific histone variants such as H3.3, are involved in the maintenance of open chromatin in ES cells (90).

Cancer is another cellular state in which global chromatin rearrangement is observed. In fact, alterations in nuclear morphology are one of the characteristics of cancer cells. Tumor-originated cells accumulate genetic and/or epigenetic differences compared with nontumor cells, and chromatin is reorganized leading to altered gene expression programs and higher plasticity. The hallmark of cancer is dedifferentiation and gene dysregulation. DNA methylation and histone modifications are two epigenetic mechanisms that are altered in cancer cells. Moreover, large organized chromatin K (lysine) modifications are reduced in cancer (91), and genes encoding proteins of the nuclear membrane present altered expression in many cancer types (92), indicating that LADs might be partially disorganized in cancer in accordance with the large-scale chromatin decondensation. Thus, it is conceivable that the distribution of histone H1 variants could be different in such reorganized nuclei, to that observed in nonmalignant cells. In turn, this could be the reason why in our study most of the H1 variants in genome regions were found to be associated with more active and open chromatin. Moreover, given the association of H1 with LADs reported here and by Izzo et al., we hypothesize that H1 could be a key player in establishing LADs in normal cells, and could also participate in the rearrangement of such domains in cancer cells due to a different prevalence of H1 variants within these domains. Alternatively, LAD reorganization in cancer cells could cause H1 variant redistribution in these genomic domains.

Tumor cells are characterized by a different methylome from that of normal cells [reviewed in (93)]. There is both global CpG hypomethylation, causing genomic instability, and hypermethylation of particular promoters including tumor-suppressor genes. In our analysis, we found that CpG islands contain H1.0, H1X and to a lesser extent H1.4, but not H1.2. This might reflect the relative abundance of these variants at promoters and suggests that promoter occupancy by H1 variants other than H1.2 is more permissive for transcription regulation in breast cancer cells. Alternatively, as H1.2 prevalence in intergenic CpG islands is also lower than that of other variants, we cannot rule out a direct role of the different H1 variants in CpG island regulation in breast cancer cells.

Similarly, within a long region of genomic sequence, genes are often characterized by having a higher GC content than the background GC content of the entire genome. We found that H1 variants except H1.2 are associated with higher GC content regions, consistent with the preferential location of H1-enriched regions within genes. H1.2 presents an inverse correlation with GC content at a genome-wide level and H1.2-enriched regions associate with lower GC content than other variants. In our analysis, H3 also associates preferentially with higher GC-content regions, in agreement with reports describing greater nucleosome-space occupancy coinciding with active transcription and higher GC contents (94).

Altogether, it seems that H1 variants are differentially associated with CpG islands and GC content in breast cancer cells. Our data are not completely consistent with previous reports showing low amounts of H1 in CpG islands (65,95). However, mouse H1d was more closely associated with GC-rich regions than H1c in the study of Cao *et al.* (65). Additionally, another study showed H1 variant-dependent interaction with DNMTs (96). In that study, it was found that, unlike other H1 variants, H1c (H1.2) does not interact with DNMT1 and DNMT3B. Based on the differential association of H1 variants with CpG islands and GC-rich regions in T47D breast cancer cells, we hypothesize that a redistribution of most of histone H1 variants in cancer may help to establish a differential chromatin state, but also an altered methylation pattern. In fact, H1 variants are differentially related to several types of cancer (33,97). Additionally, comparison of human mammary epithelial cells with breast cancer cell lines including T47D (98) showed global massive hypomethylation at CpG-poor regions, and hypermethylation at CpG-rich gene-related regions, proximal to the TSS, where local enrichment of all H1 variants except H1.2 is observed in our data. Moreover, hypomethylated regions in breast cancer cells coincide with repressive chromatin, gene silencing, repressive histone posttranslational modifications (PTMs), intergenic regions and LADs (99), which in turn coincides with an enrichment of H1.2 found in our analysis. Further investigation of the DNA methylation profile of T47D breast cancer cells could confirm a differential role of H1 variants in establishing or maintaining DNA methylation in breast cancer.

Chromatin containing H1 variants other than H1.2 might support a level of compaction that facilitates a rapid conversion into either an active or a repressed state and, consequently, these variants are allowed at TSS of genes before activation. In fact, a particular posttranslational modification in H1.4 (K34Ac) has been found to locate around the TSS of active genes (49). Instead, we have described that H1.2 occupancy at distal promoters is the best predictor of gene repression. Moreover, genes presenting H1.2-enriched regions are clearly less strongly expressed than average. This study points toward the inclusion of H1.2 as a repression mark and to it being associated with closed chromatin. In this regard, H1.2 has been found to be included in a p53-containing repressive complex in HeLa cells (50), and murine H1.2 has been found to be developmentally upregulated in the retina, promoting facultative heterochromatin formation in mature rod photoreceptors (100).

Several studies have compared the chromatin binding affinity and residence time on chromatin of the different H1 subtypes in different organisms or cell lines, as well as its nuclear localization, obtaining diverse, if not controversial, results on the functional heterogeneity of H1 variants. In general, H1.2 is among the variants presenting intermediate or low affinity for chromatin and, consequently, elevated mobility. Instead, H1.4 has been mostly associated with high affinity, low mobility and colocalization with heterochromatin (40,101–103). We do not fully understand how these properties may relate or contradict our observation of H1.2 being enriched in repressed and gene-poor chromatin in breast cancer cells. Certainly, different experimental approaches performed in the same cell model would facilitate to reconcile the different observations.

There is nowadays increasing evidence of a 3D organization of the genome within the cell nucleus. Interphase chromatin is organized in large chromosome territories defined as ‘topological domains’, which can interact despite being several megabases apart (104,105). These domains are stable across different cell types and highly conserved across species. It has already been reported that embedded genes in these domains are in a transcriptionally similar state and associated with transcriptionally related histone marks and chromatin features. Hence, it is not unreasonable to speculate that H1 could be involved in the formation or maintenance of such domains due to its role in chromatin structure. High-throughput profiling of chromatin marks and components has recently made it possible to define chromatin states (106,107). In *Drosophila* cells, five principal chromatin types have been described, H1 being present in all of them in different proportions (107). Although this may reflect the general features of H1 occurrence, in cells presenting several H1 subtypes a differential distribution of subtypes between different chromatin types may occur, as is suggested in our study. We have found that H1.2 is the variant most closely associated with LADs, low GC content and gene-poor regions and chromosomes that are normally located at the periphery of the nucleus, features related to chromatin compaction, while chromatin associated with the other variants presents features of a more plastic chromatin. Interestingly, gene-rich chromosomes, presumably with a more dynamic chromatin and histone H1 exchange, and located toward the center of the nucleus, are enriched in H1 variants synthesized all through the cell cycle, namely H1.0 and H1X. It would be interesting to further analyze the colocalization of the different human H1 variants with chromatin marks and components that better define the diverse chromatin states, although these types of comparisons are limited by the availability of high-throughput data on the same or related cell types.

## ACCESSION NUMBERS

The data sets are available in the Gene Expression Omnibus (GEO) database under the accession number GSE49345.

## SUPPLEMENTARY DATA

Supplementary Data are available at NAR Online.

## FUNDING

Ministerio de Ciencia e Innovación of Spain (MICINN) and FEDER [BFU2011-23057 to A.J.]; Ministerio de Economía y Competitividad [SAF2012-36199 to N.L.-B.]; Generalitat de Catalunya [2009-SGR-1222 to A.J.]; JAE-Doc contract from CSIC-MICINN (to J.-M.T.); TA contract from CSIC-MICINN (to R.M.); FPU predoctoral fellowship from MICINN (to L.M.-A.). Funding for open access charge: Ministerio de Ciencia e Innovación of Spain (MICINN) and FEDER [BFU2011-23057 to A.J.].

*Conflict of interest statement*. None declared.

## Supplementary Material

Supplementary Data
