# Supplementary material for: Mapping of six somatic linker histone H1 variants in human breast cancer cells uncovers specific features of H1.2
Source: Nucleic Acids Res. 2014 Jan 28;42(7):4474–93. doi: 10.1093/nar/gku079 (PMC3985652; doi:10.1093/nar/gku079)
Supplement: Supplementary Data [file supp_42_7_4474__index.html]

Mapping of six somatic linker histone H1 variants in human breast cancer cells uncovers specific features of H1.2 — Mapping of six somatic linker histone H1 variants in human breast cancer cells uncovers specific features of H1.2 — Supplementary Data 

# Mapping of six somatic linker histone H1 variants in human breast cancer cells uncovers specific features of H1.2

## Supplementary Data

files

**Files in this Data Supplement:**

- Supplementary Data - pdf file
