# Supplementary material for: Mapping of six somatic linker histone H1 variants in human breast cancer cells uncovers specific features of H1.2
Source: Nucleic Acids Res. 2014 Jan 28;42(7):4474–93. doi: 10.1093/nar/gku079 (PMC3985652; doi:10.1093/nar/gku079)
Supplement: Supplementary Data [file supp_gku079_nar-03245-m-2013-File009.pdf]

**Supplementary Data**

**Figure S1.** Expression of HA-tagged somatic histone H1 variants in breast cancer cells. Chromatin was prepared from T47D-derived cells stably expressing HA-tagged H1 variants, wild-type or a K26A mutant of H1.4, and loaded into a 10% SDS-PAGE. Western blot hybridization was performed with anti-HA antibody (A), or with H1 variant-specific antibodies (B).

**Figure S2.** Specificity of the anti-HA antibody. ChIP was performed in cells expressing H1-HA or mock infected with the empty retroviral expression vector with anti-HA antibody or unrelated immunoglobulins (IgG), and the abundance of IPed material was quantified by qPCR with ß-actin promoter oligonucleotides. A representative experiment performed in triplicate is shown.

**Figure S3.** HA-tagged H1 variants are associated with gene promoters, coding regions and repetitive DNA. ChIP was performed in cells expressing H1-HA with anti-HA antibody and the abundance of IPed material was quantified by qPCR with oligonucleotides for the indicated promoters, coding or heterochromatic regions, and corrected by input DNA amplification with the same primer pair. A representative experiment performed in triplicate is shown.

**Figure S4.** Specificity of H1.2 and H1X antibodies in ChIP experiments determined in H1 variant-specific knock-down cells. T47D-derived cells stably harboring an inducible system for shRNA expression against H1.2 or H1X expression, were treated with doxycycline for 6 days or left untreated. Then, ChIP was performed with H1 variant-specific antibodies against H1.2 and H1X (or control IgG), and the IPed material was quantified by qPCR with oligonucleotides for the indicated promoters (-10 kb distal promoter or TSS), and corrected by input DNA amplification. A representative experiment performed in triplicate is shown. Western blot hybridizations showing the rate of H1 depletion upon doxycycline treatment are shown.

**Figure S5.** An H1 valley at TSS is found at genes being expressed that show FAIRE-measured open chromatin and increased H3K4me3 at TSS. Several genes representing different levels of expression according to microarrays data were chosen to analyze

mRNA abundance by RT-qPCR, chromatin accessibility at TSS by FAIRE-qPCR, and distribution of H1, H3 and H3K4me3 by ChIP at distal (upstream) promoter compared to TSS. ND, not determined. RT-PCR values for each gene were normalized to GAPDH expression and genomic DNA amplification with the same set of primers. ChIP and FAIRE PCRs were normalized to input DNA. A representative experiment performed in triplicate is shown.

**Figure S6.** H1 depletion at TSS of hormone-responsive promoters upon stimulation with ligand. T47D-derived cells harboring an MMTV-luciferase construct and expressing different H1-HA were treated with a progestin (R5020 10nM) for the indicated time and ChIP was performed with anti-HA antibody. The abundance of IPed material was quantified by qPCR with specific oligonucleotides for the MMTV promoter (nucleosome B). A representative experiment performed in triplicate is shown.

**Figure S7.** H1 depletion at TSS of inducible promoters. The H1 valley at TSS of JUN and FOS genes is preformed and increases upon mitogenic stimulation. T47D cells were treated with PMA 100nM for 60 min or left untreated and ChIP was performed with H1, H1.2, H1X and H3 antibodies. The abundance of IPed material was quantified by qPCR with oligonucleotides for the indicated promoters (-10 kb distal promoter or TSS), and corrected by input DNA amplification. JUN and FOS were responsive to PMA as shown in the RT-qPCR experiment (right panels). PSMB4 and OCT4 are non-responsive control genes, active and repressed, respectively, in T47D cells. The table below shows the H1 valley ratio (calculated as distal/TSS) at 0 and 60 min, or the relative H1 valley ratio at 60 min compared to 0 min. A representative experiment performed in triplicate is shown.

**Figure S8.** Global gene expression profile in T47D cells. (A) Overlap between unique transcript IDs at the Nimblegen promoter array and Agilent expression microarray. (B) Gene expression profile in T47D cells of 62,976 transcripts, from highest to lowest expression, obtained by hybridization with an Agilent microarray.

**Figure S9.** Some repressed genes show an H1.2 valley at the TSS. ChIPed material from H1.0-HA cells or with the specific H1.2 antibody was quantified by qPCR with oligonucleotides for the indicated promoter regions and corrected by input DNA

amplification. Selected genes belong to the indicated expression profile percentiles. A representative experiment performed in triplicate is shown.

**Figure S10.** Non protein-coding transcripts show an H1.2 valley at TSS. (A) Heat maps of ChIP-chip probe intensity around TSS (-3200 to +800 bp) for 1,145 non protein-coding transcripts (NRs) from which the expression rate was determined (B). NRs are ordered from highest to lowest gene expression. (B) Expression levels of NRs are shown as a box plot compared to total transcriptome included in the Agilent expression microarray. Significance was tested using the Kolmogorov-Smirnov test. p-value < 2.2e-16.

**Figure S11.** Correlation between the abundance of different H1 variants at distal promoter regions.

(A) Heat map and dendrogram of the Pearson's correlation coefficient between all H1 variants ChIP-chip samples.

(B) Scatter plots of the abundance of different H1 variants at distal promoter regions. X and Y axis represent mean probe intensity at distal promoter regions (-3200 to -2000 bp relative to TSS), for the indicated H1 variants. R: Pearson's correlation coefficient.

**Figure S12.** Coincidence between genes presenting the highest or lowest H1.2 or H1X content at distal promoter. (A) Heat maps of H1 ChIP-chip probe intensity around TSS. Genes are ordered from lowest to highest H1.2 (left) or H1X (right) content at distal promoter regions. Genes with the top or lowest distal H1 content are indicated. These genes (2050 genes for each group, 10% of the total) were used to determine the number of coinciding genes as shown in Venn diagrams (B). A streaking coincidence exist between genes presenting few H1.2 but high H1X.

(C) Expression levels of coinciding genes in the four comparisons depicted in (B). Genes were classified in five expression groups (EG) similar to Figure 2, and the percentage of coinciding genes belonging to each of these groups was determined. (Right panel) Expression levels of coinciding genes is also shown as a box plot. Significance was tested using the Kolmogorov-Smirnov test. Enrichment and depletion is marked with red and blue asterisks, respectively. ** p-value<0,001 and * p-value<0,005.

**Figure S13.** Coincidence between genes presenting highest or lowest H1.2 or H1.0-HA content at distal promoter. See Figure S12 legend.

**Figure S14.** The ratio between H1.2 and H1X abundance at selected genes is conserved among T47D and HeLa cells, but not in MCF7, in relation to the cell abundance of each variant.

(A) ChIP-qPCR of H1.2 and H1X abundance at TMEM204, TUBGCP5, COL4A3 and CUGBP2 distal promoter regions in T47D, HeLa and MCF7 cells. A representative experiment performed in triplicate is shown.

(B) Abundance of H1 variants in T47D, HeLa and MCF7 chromatin determined by immunoblot with specific antibodies.

(C) Expression of H1 variants in T47D, HeLa and MCF7 cells determined by RT-qPCR. cDNA levels for the indicated H1 variants were corrected by GAPDH expression and amplification of genomic DNA with the same PCR primers. A representative experiment performed in triplicate is shown.

**Figure S15.** Comparison of H1.2 and H1X promoter content among T47D and HeLa cell lines. Heat maps of H1.2 and H1X ChIP-chip probe intensity around TSS (-3200 to +800 bp) for 20,338 transcripts. Genes in all heat maps shown are ordered from lowest to highest H1.2 content at distal promoter regions in T47D cells.

**Figure S16.** H1 variant abundance around TSS for genes ordered according to their position along selected human chromosomes.

(A) Cluster dendrogram of all human chromosomes depending on their gene richness coefficient and gene expression. The gene-richness coefficient for each chromosome was calculated as described in Figure 4. Average gene expression for individual chromosomes was obtained from microarray data obtained in T47D cells. GRC$\geq$2 are shown in the same color.

(B) Heat maps of H1.2, H1.0-HA and H1X ChIP-chip probe intensity around TSS (-3.2 to +0.8 kbp) for genes ordered according to their position along selected gene-poor or gene-rich chromosomes in T47D cells.

(C) Heat maps of H1.2 and H1X ChIP-chip probe intensity around TSS (-3.2 to +0.8 kbp) for genes ordered according to their position along chromosome 1 in T47D and HeLa cells. Heat map of H1.0-HA in T47D is also included.

**Figure S17.** Occupancy of H1 variants at regions enriched for different genome features and histone marks.

(A) Box plots showing the occupancy of H1 variants (average, input-subtracted ChIP-Seq signal) at regions enriched for the indicated genome features: DNase hypersensitivity sites (data from T47D cells), FAIRE regions (HeLa data), CTCF and p300 binding sites (T47D data).

(B) Box plots showing the occupancy of H1 variants (average, input-subtracted ChIP-Seq signal) at regions enriched for the indicated histone marks (data from HeLa cells).

(C) Box plots showing the occupancy of H1 variants (average, input-subtracted ChIP-Seq signal) at CpG islands. Significance was tested using the Kolmogorov-Smirnov test taking as a control a random sample of windows with equal width to the indicated histone mark. Enrichment and depletion is marked with red and blue asterisks, respectively. * p-value<0,001.

**Figure S18.** Distribution of H1 variants along selected chromosome regions containing LADs. Input-subtracted H1 variants and H3 ChIP-seq data viewed in the UCSC genome browser together with GC content, RefSeq genes, H3K4me3 (ENCODE average of 9 cell lines), CpG and LADs.

**Figure S19.** Distribution of H1 variants along the entire length of chromosomes 3, 4, 10, 13, 17 and 21. Input-subtracted H1 variants and H3 ChIP-seq signal viewed in the UCSC genome browser together with GC content, RefSeq genes, H3K4me3 (ENCODE average of 9 cell lines), CpG and LADs.

**Figure S20.** GC content of H1-enriched chromatin. (A) Genome-wide correlation scatter plots of H1 variants versus GC content. X axes: average input-subtracted H1 variants and H3 ChIP-seq signal (normalized to 1000bp window). Y axes: GC%. R: Pearson's correlation coefficient. (B) Box plot of the percentage of GC in regions found enriched and depleted for each H1 variant.

**Figure S21.** Correlation scatter plots between the occupancy of H1 variants at all chromosomes and the gene richness coefficient or average gene expression. Correlation

between gene richness coefficient and gene expression of all chromosomes is also shown. R: Pearson's correlation coefficient.

**Figure S22.** Annotation of H1 enriched and depleted regions to promoters, genes and intergenic regions and overlap with CpG islands. (A) H1 variants enriched and depleted regions over input were mapped to promoters (defined as -5 kb to +1 kb from TSS), genes (-5kb from TSS to +3kb from TTS) or intergenic regions (rest of the genome). The percentages of identified regions for the different variants falling into each of these three categories are represented. Notice that 100% is the sum of genic and intergenic regions. The theoretical occupancy of these compartments in the UCSC genome is shown as percentages, as well as occupancy of total ChIP-seq input reads (right panel).
(B) Co-localization of H1 enriched or depleted regions with CpG islands expressed as percentage of total H1 regions overlapping CpG sites. Areas of enrichment or depletion of H1 variants compared to input derived from ChIP-seq data with a fold-change $\geq 2$ were considered.

**Figure S23.** Co-localization of H1 enriched regions with CpG islands. Distribution of H1 variants along selected regions of the genome containing CgG sites and H1 enriched regions identified using SICER software. Input-subtracted H1 variants and H3 ChIP-seq signal viewed in the UCSC genome browser together with GC content, RefSeq genes, H3K4me3 (ENCODE average of 9 cell lines) and CpG islands.

**Figure S24.** Venn diagrams showing the overlap between genes containing enriched or depleted regions of the different H1 variants. H1 enriched or depleted regions mapping within genes (-5kb from TSS to +3kb from TTS) were used to identify target genes. Comparisons between different H1 variants are shown in two clusters for clarity coinciding with different ChIP-seq experiments. The expression profiles of target genes containing enriched or depleted regions for a unique variant are shown as box plots in Figure 7.

**Figure S25.** Genomic annotation of enriched or depleted regions of individual H1 variants. Pie diagram of the distribution of H1 variants enriched regions at genes, proximal regulatory regions, and distal intergenic regions. Promoter and downstream regions are defined as 3,000bp upstream TSS or downstream TTS, respectively. As a

control, the annotation of all genome base pairs is shown. Two replicas of endogenous H1.2 ChIP-seq experiments are shown.

**Table S1.** Summary of samples analyzed by ChIP-seq in three independent experiments (r1, r2, r3; replica 1, 2 and 3). Read length, number of total reads obtained, and number of mappable reads to the human genome version hg18, as well as mapped rate, are shown.

**Table S2.** Gene ontology of genes presenting the highest (top 10%) or lowest (bottom 10%) H1.2 or H1X content at distal promoter (-3200 to -2000 bp relative to TSS) according to ChIP-chip data shown in Figure 3. P-value (adjusted for multiple testing by Benjamini method) and false discovery rate are shown.

**Table S3.** Summary of enriched or depleted regions of individual H1 variants and its target genes. Areas of enrichment or depletion of H1 variants compared to input derived from ChIP-seq data with a fold-change equal or greater than 2 were considered. Genes were defined as comprised between -5kb from TSS to +3kb from TTS, and promoters from -5kb upstream TSS to +1kb downstream TSS.

**Supplementary Methods.** Cell treatments. Antibodies. H1 extraction, gel electrophoresis and immuniblotting. FAIRE assays. RNA extraction and RT-PCR.

# Figure S1

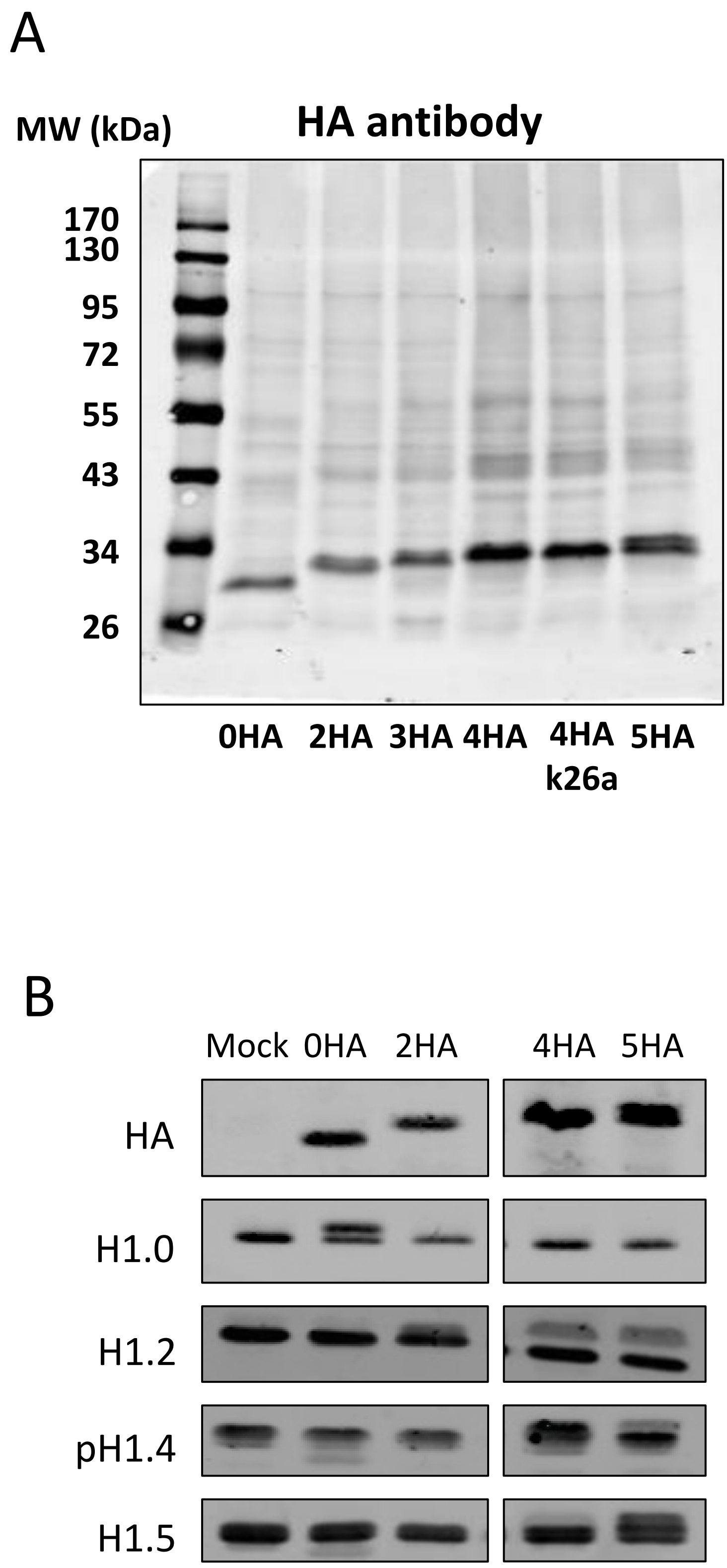


Millán-Ariño, Ll., et al.

# Figure S2

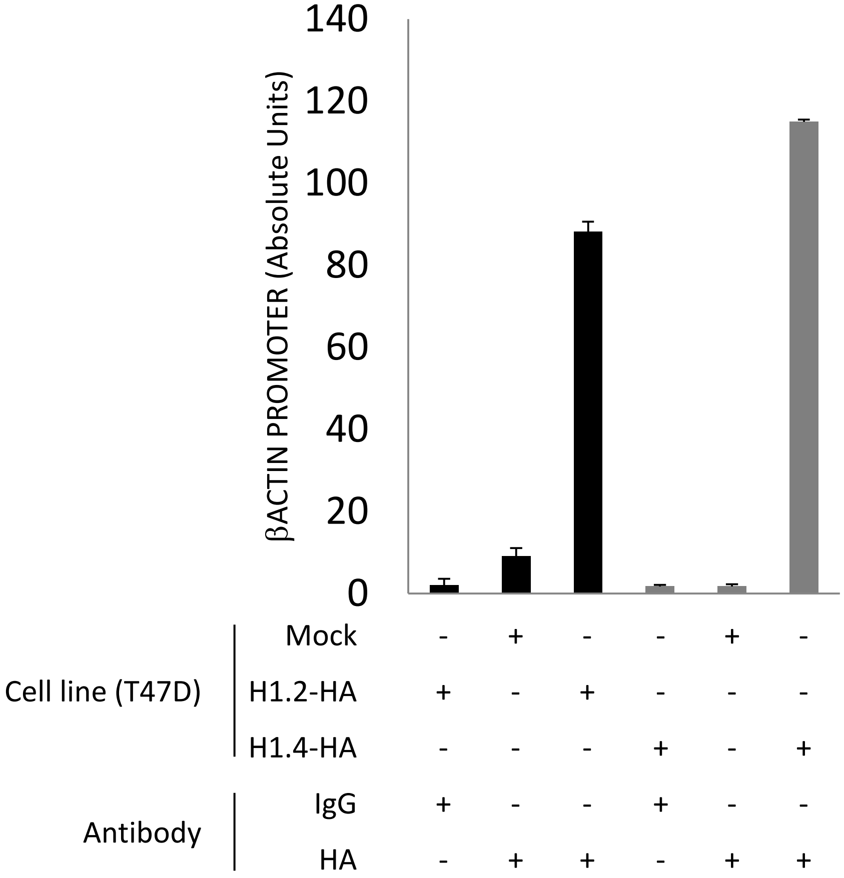


Millán-Ariño, Ll., et al.

# Figure S3

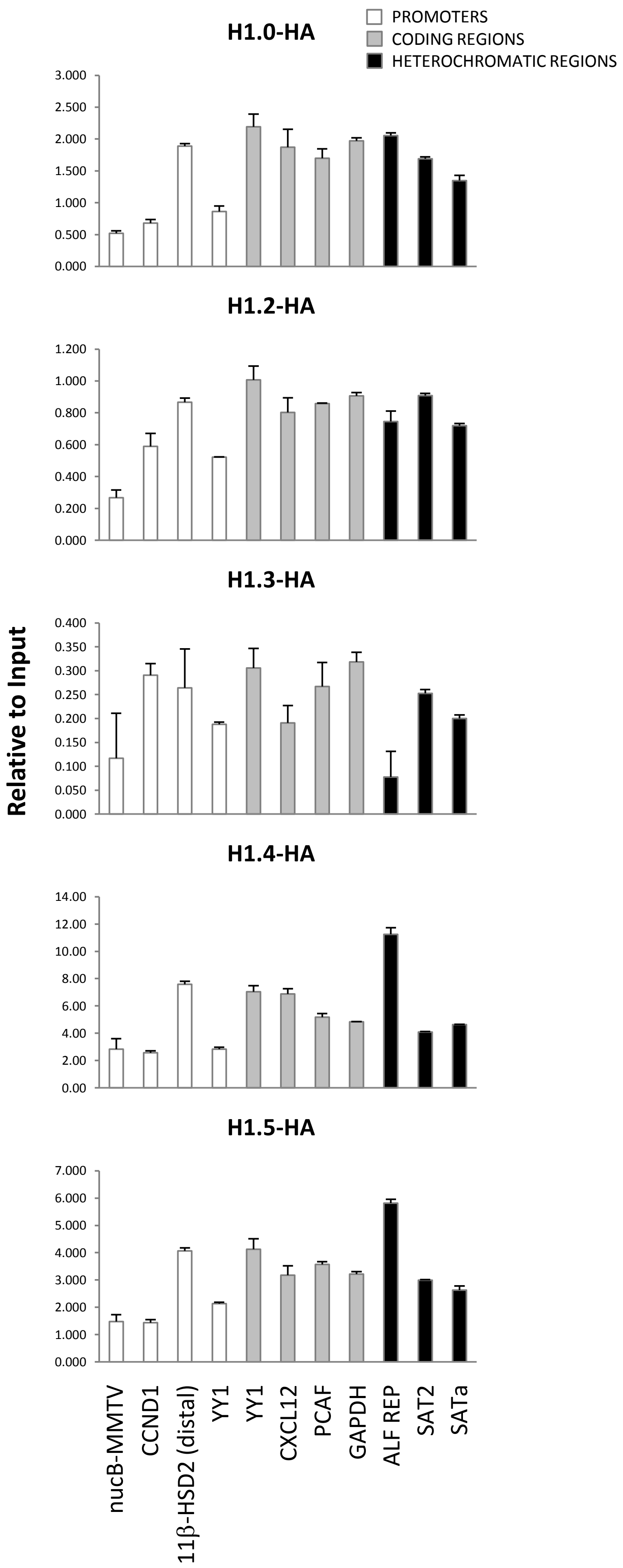


Millán-Ariño, Ll., et al.

# Figure S4

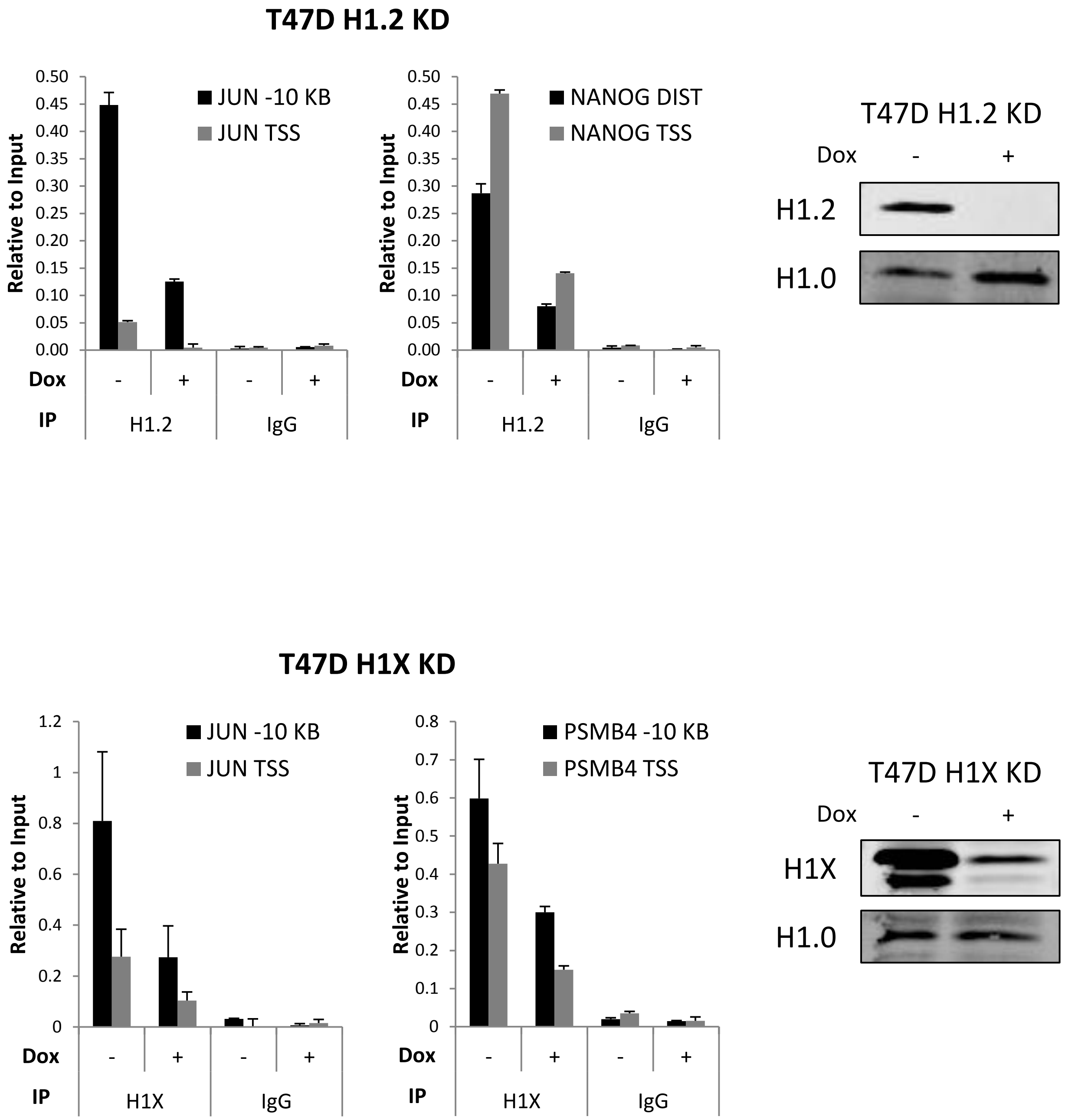


Millán-Ariño, Ll., et al.

# Figure S5

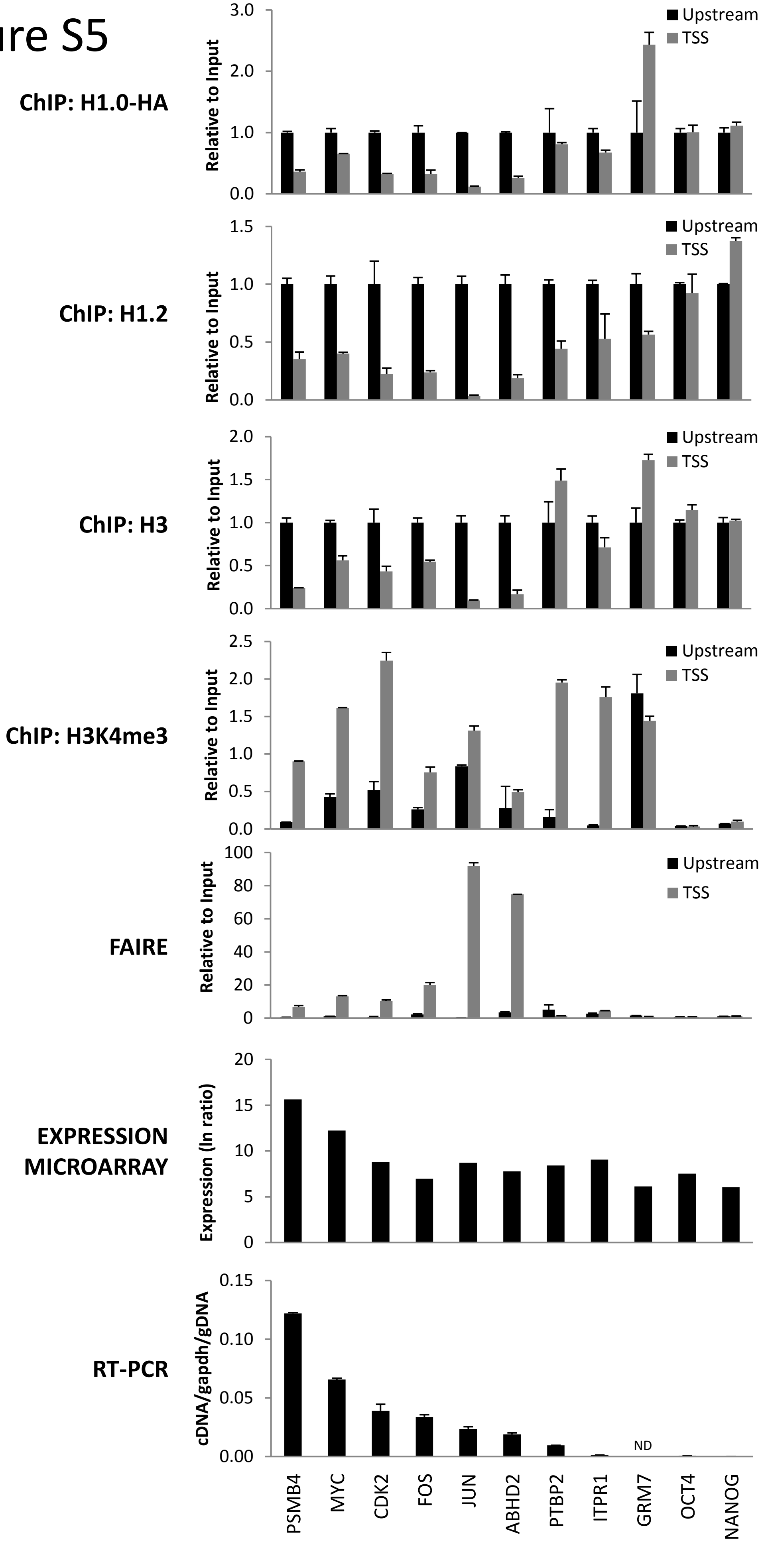


Millán-Ariño, Ll., et al.

# Figure S6

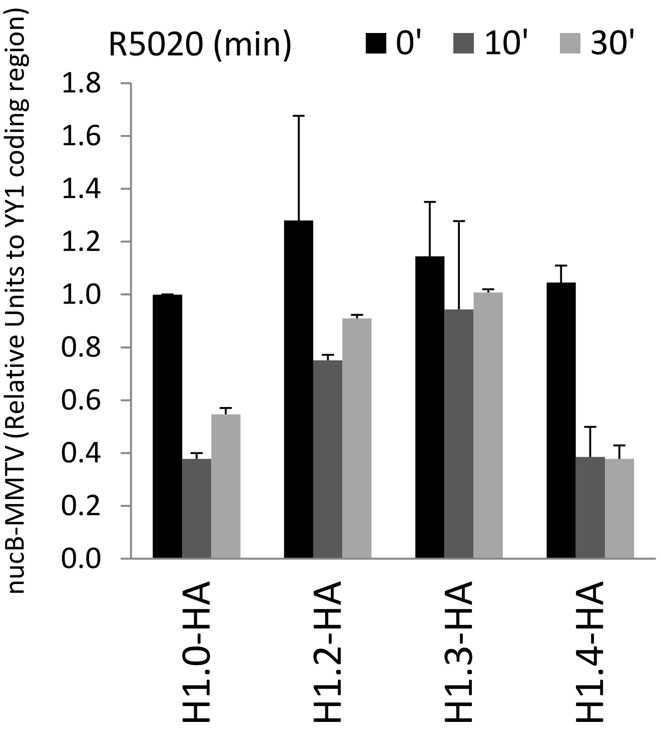


Millán-Ariño, Ll., et al.

Figure S7

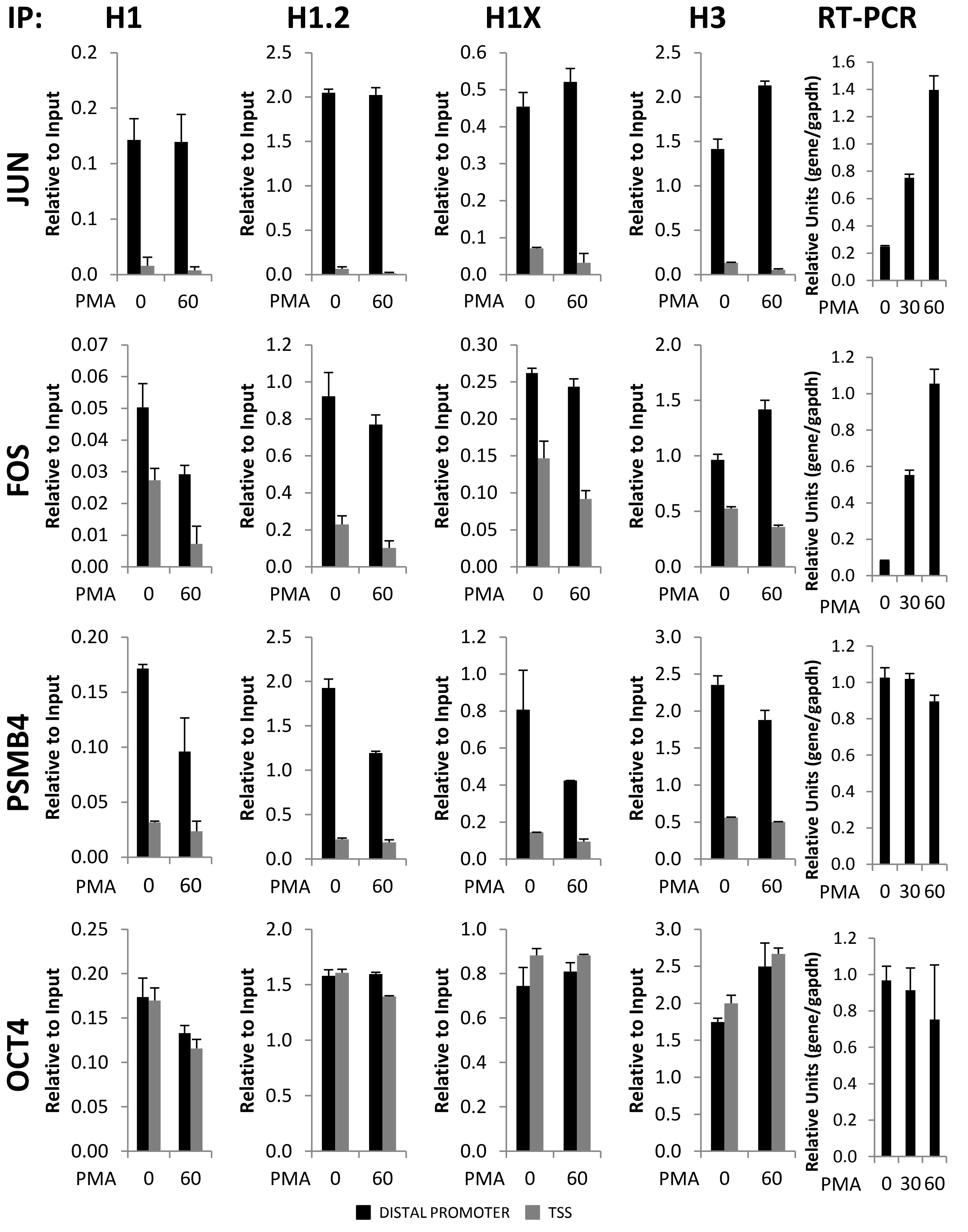


| | IP | H1 | | H1.2 | | H1X | | H3 | |
|---|---|---|---|---|---|---|---|---|---|
| | PMA (min) | 0 | 60 | 0 | 60 | 0 | 60 | 0 | 60 |
| JUN | valley Fc | 15,18 | 29,64 | 30,68 | 115,42 | 6,41 | 16,01 | 10,56 | 39,45 |
| | relative Fc | 1,00 | 1,95 | 1,00 | 3,76 | 1,00 | 2,50 | 1,00 | 3,73 |
| FOS | valley Fc | 1,84 | 4,02 | 4,01 | 7,49 | 1,79 | 2,65 | 1,83 | 3,94 |
| | relative Fc | 1,00 | 2,18 | 1,00 | 1,87 | 1,00 | 1,48 | 1,00 | 2,15 |
| PSMB4 | valley Fc | 5,45 | 4,06 | 8,70 | 6,30 | 5,59 | 4,46 | 4,20 | 3,76 |
| | relative Fc | 1,00 | 0,75 | 1,00 | 0,72 | 1,00 | 0,80 | 1,00 | 0,89 |
| OCT4 | valley Fc | 1,02 | 1,15 | 0,98 | 1,14 | 0,84 | 0,92 | 0,87 | 0,94 |
| | relative Fc | 1,00 | 1,12 | 1,00 | 1,16 | 1,00 | 1,09 | 1,00 | 1,07 |

# Figure S8

A

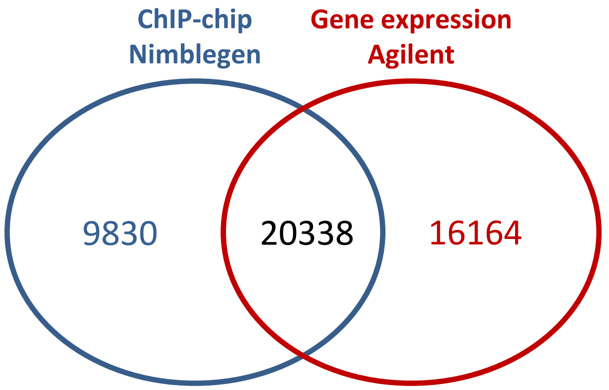


B

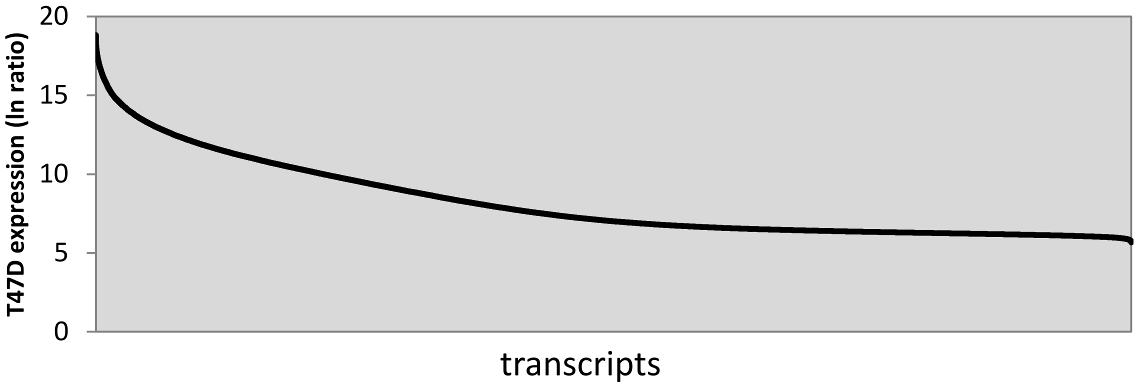


Millán-Ariño, Ll., et al.

# Figure S9

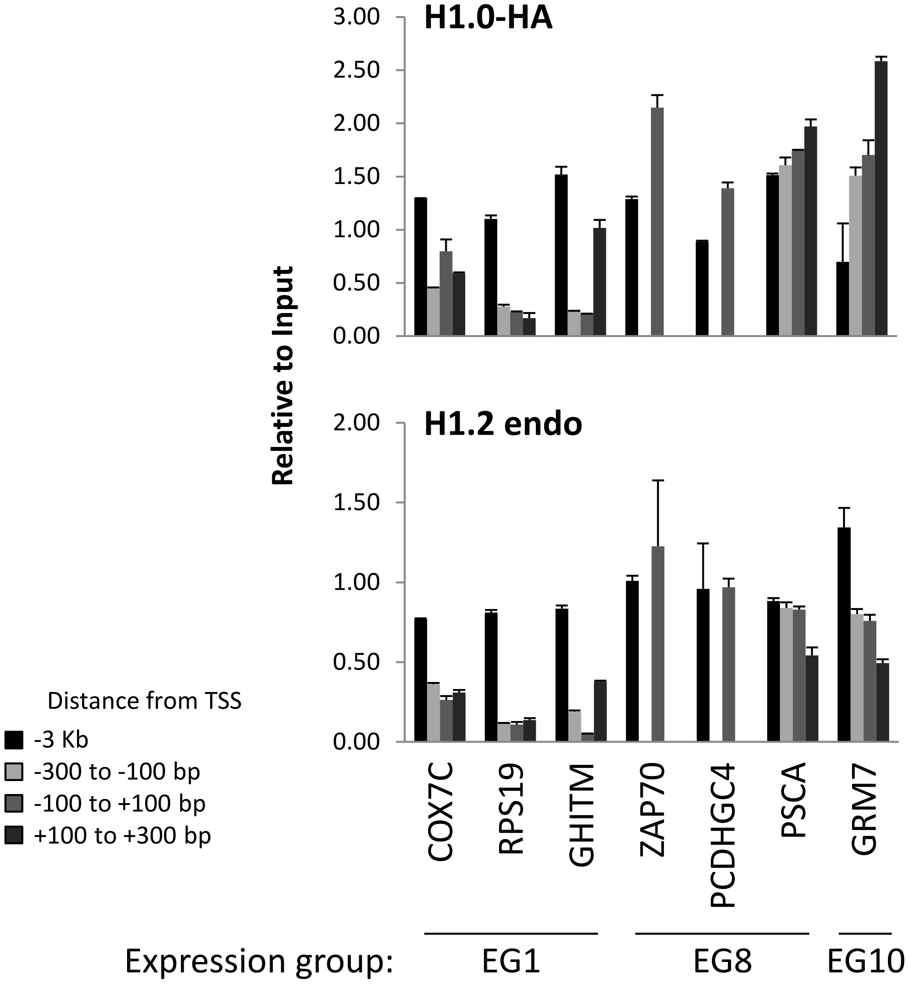


Millán-Ariño, Ll., et al.

# Figure S10

A

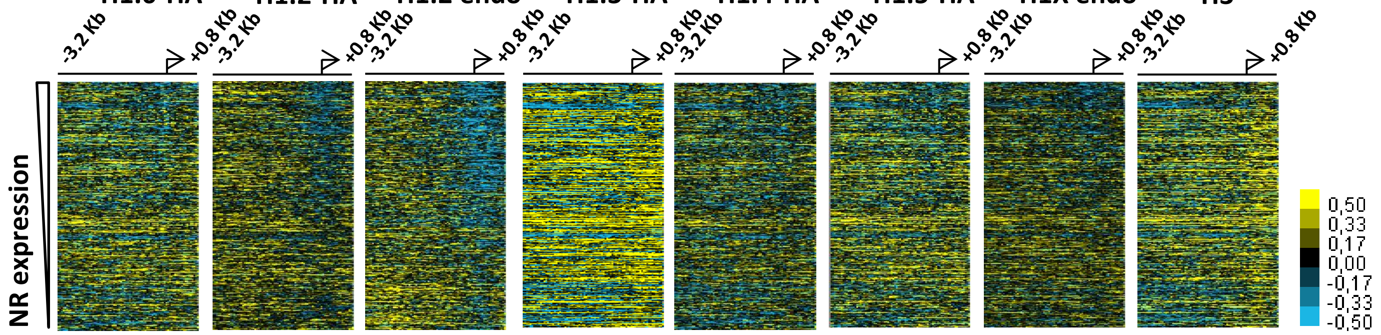


B

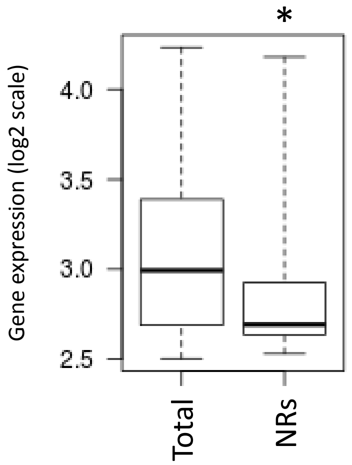


Millán-Ariño, Ll., et al.

# Figure S11

A

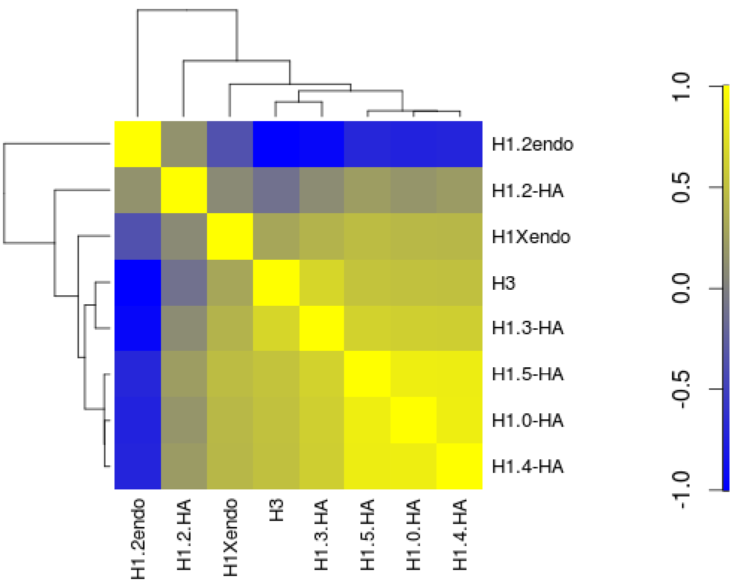


B

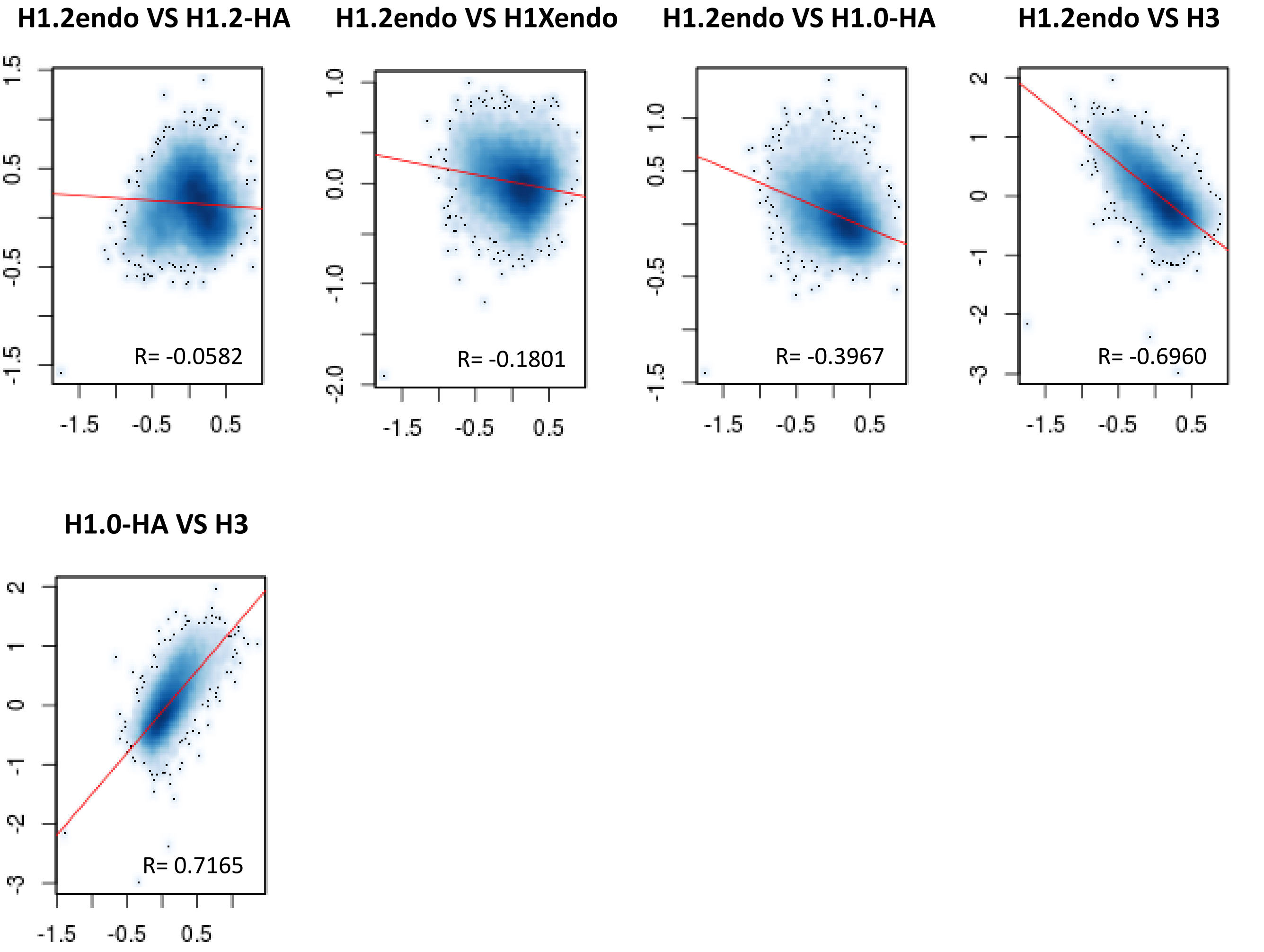


Millán-Ariño, Ll., et al.

# Figure S12

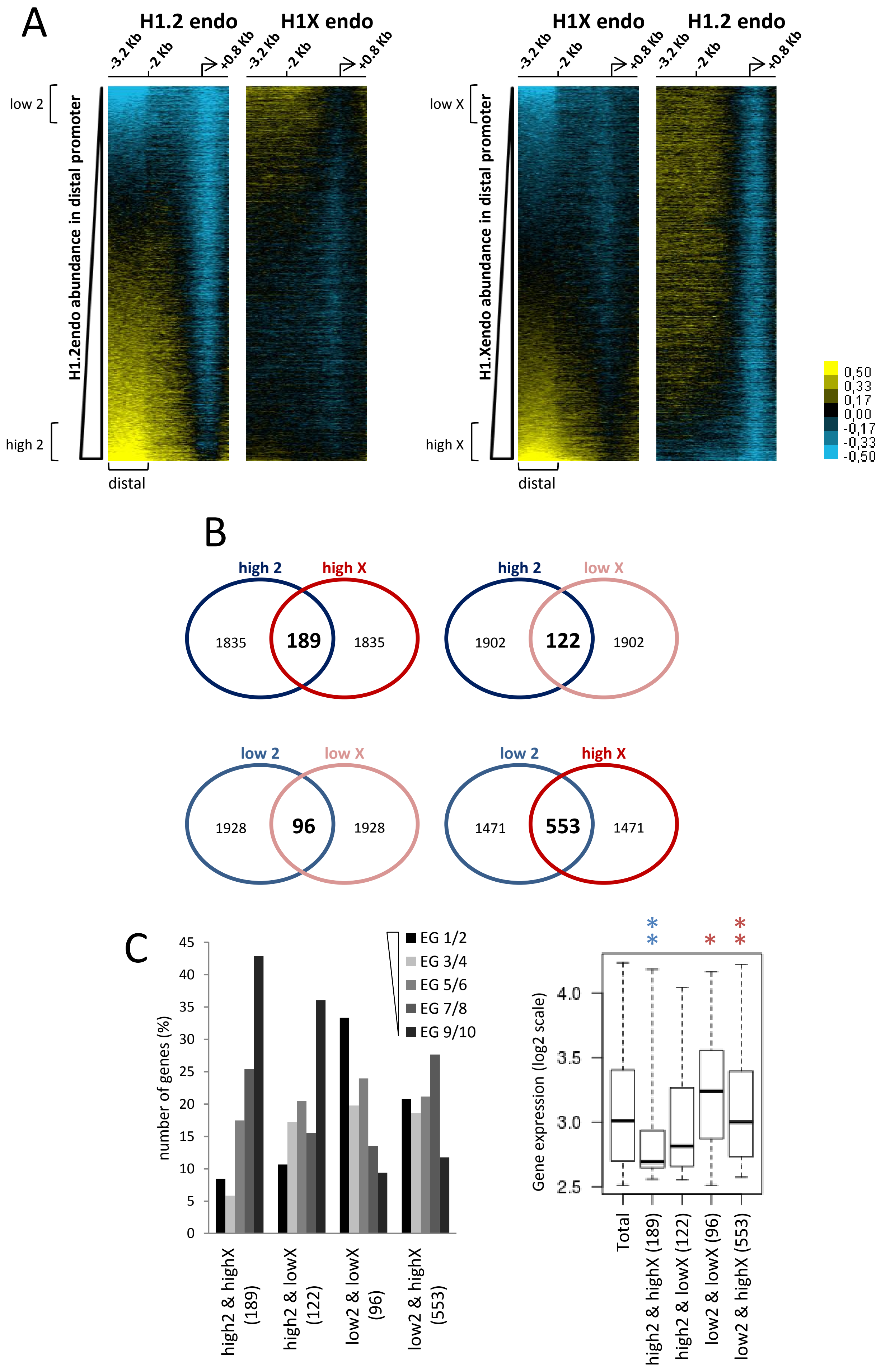


Millán-Ariño, Ll., et al.

# Figure S13

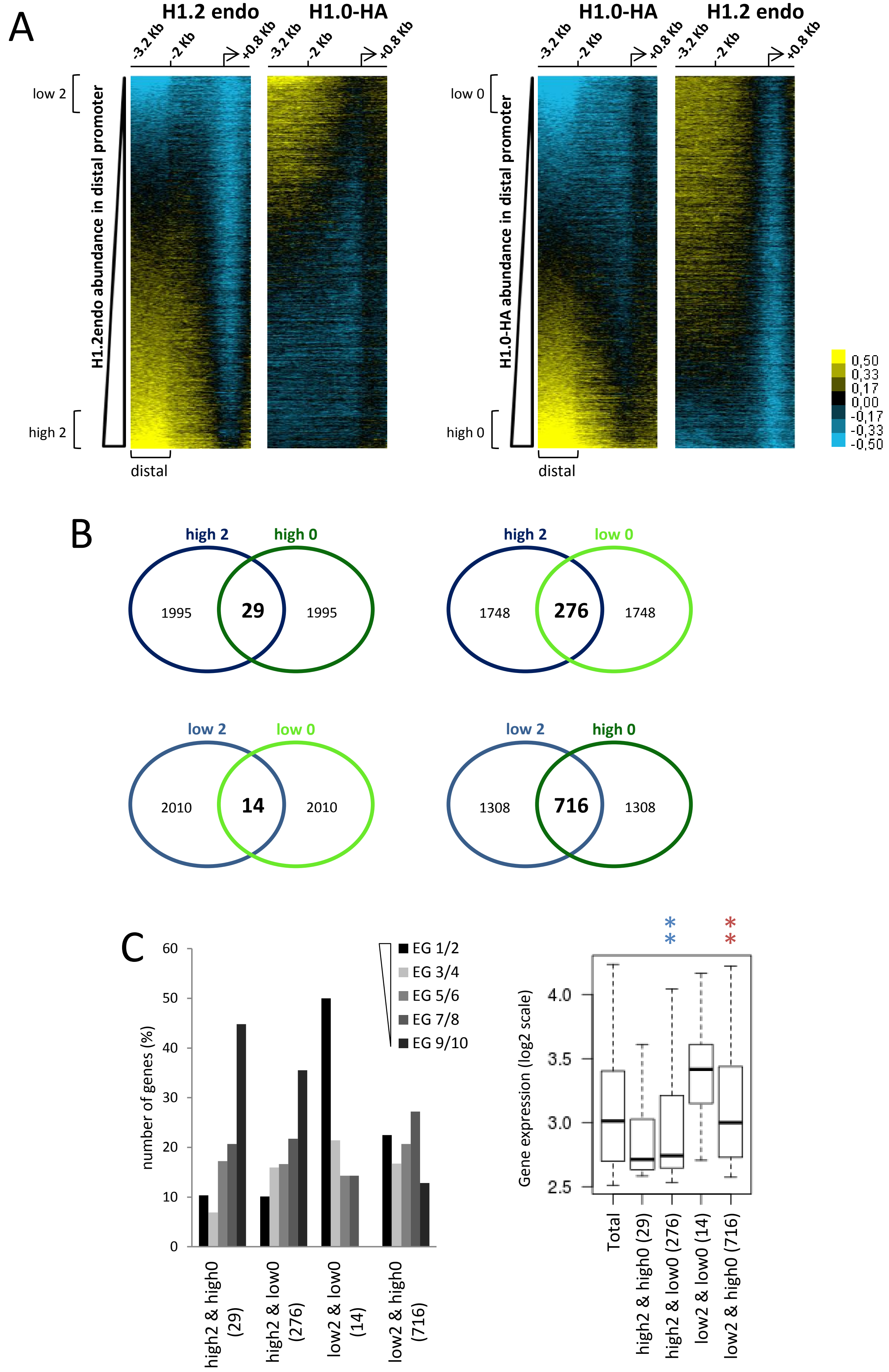


Millán-Ariño, Ll., et al.

# Figure S14

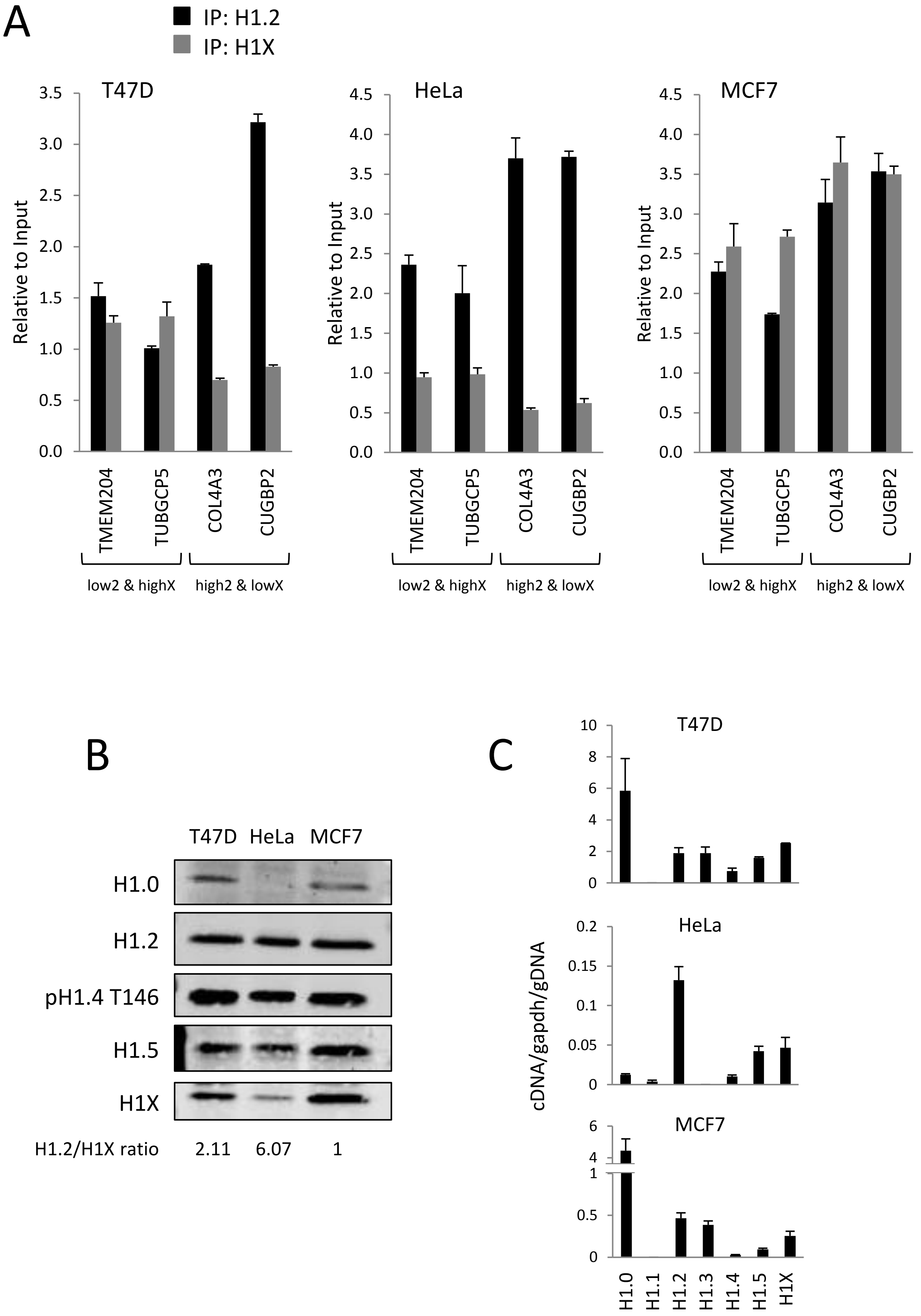


Millán-Ariño, Ll., et al.

# Figure S15

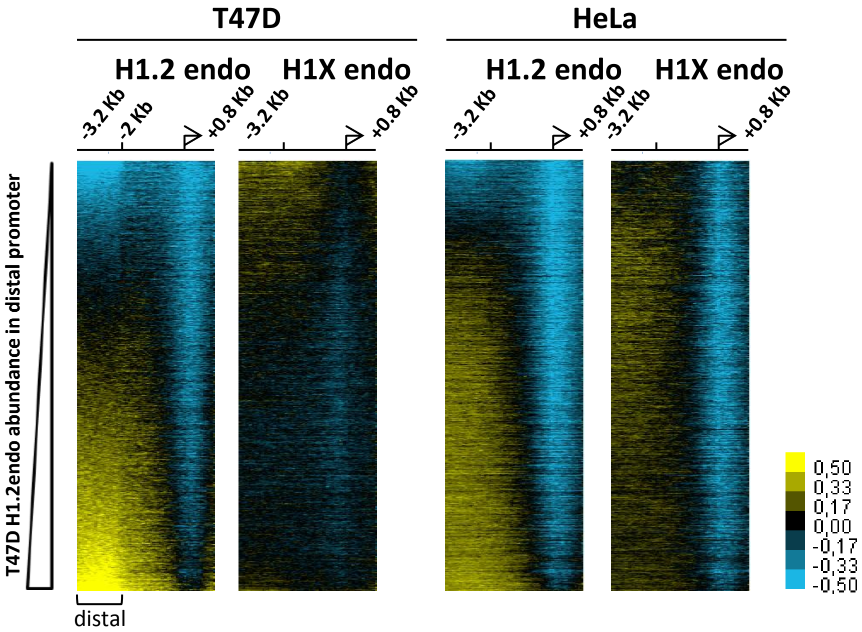


Millán-Ariño, Ll., et al.

# Figure S16

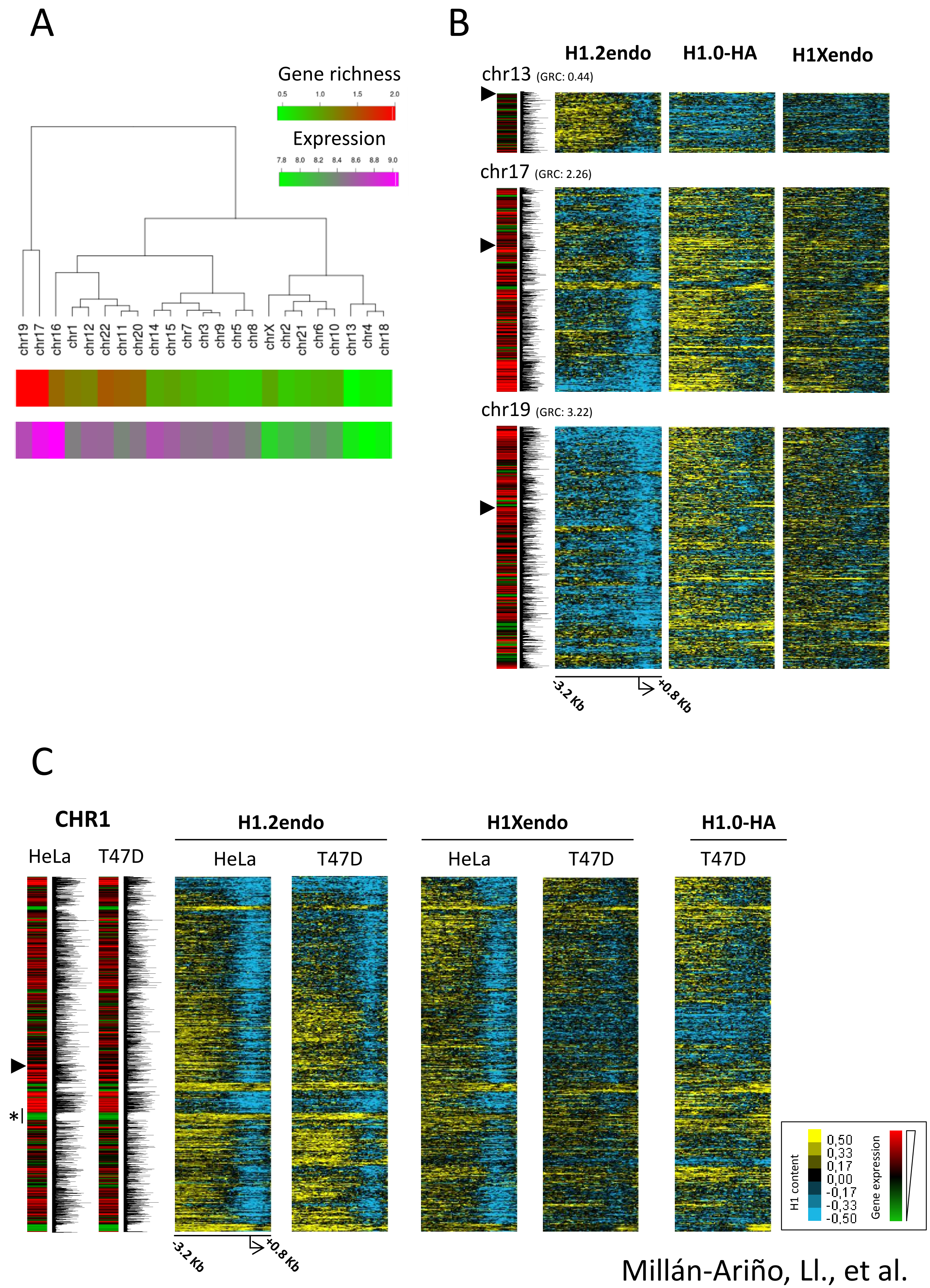


Millán-Ariño, Ll., et al.

# Figure S17

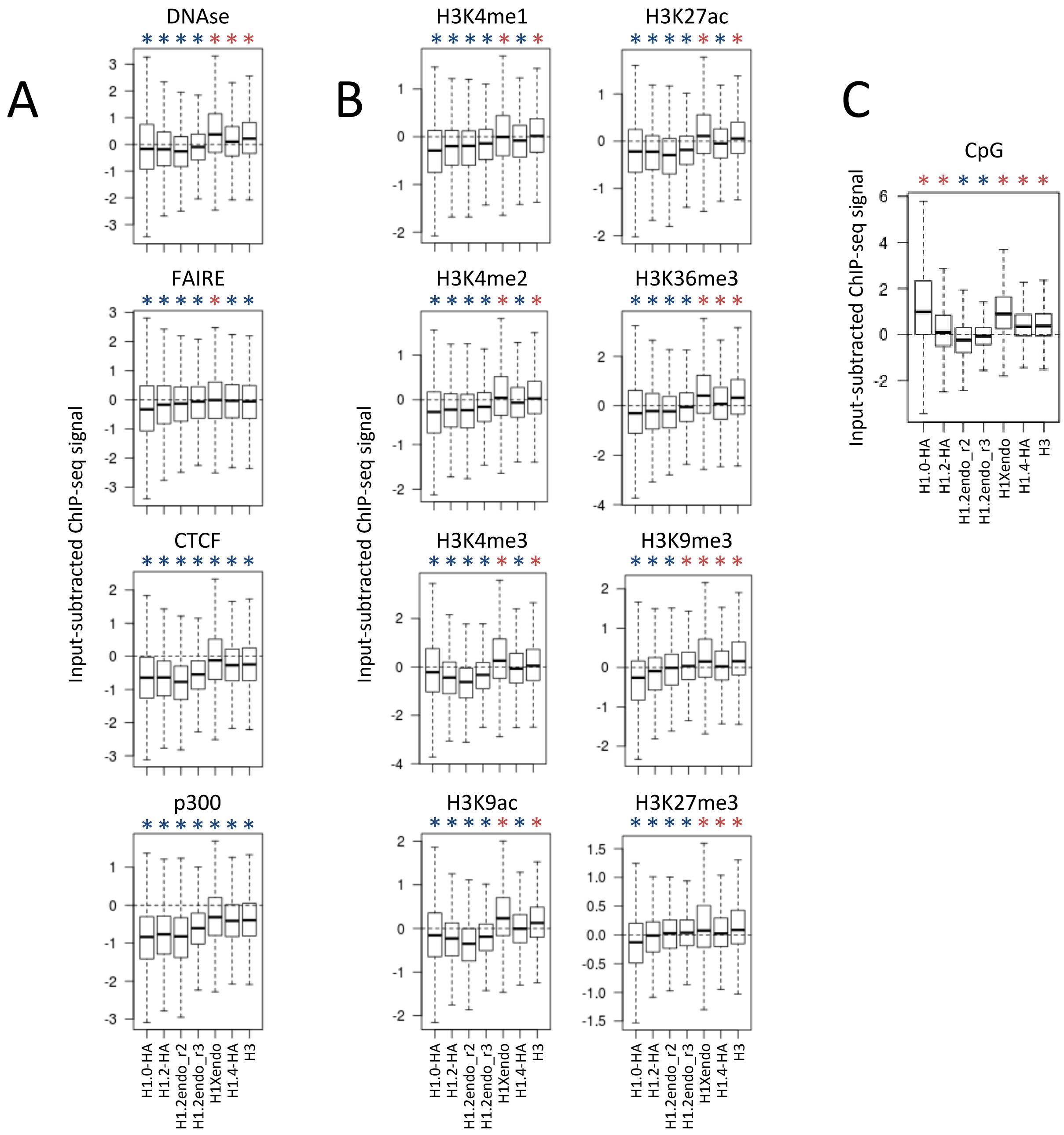


Millán-Ariño, Ll., et al.

# Figure S18

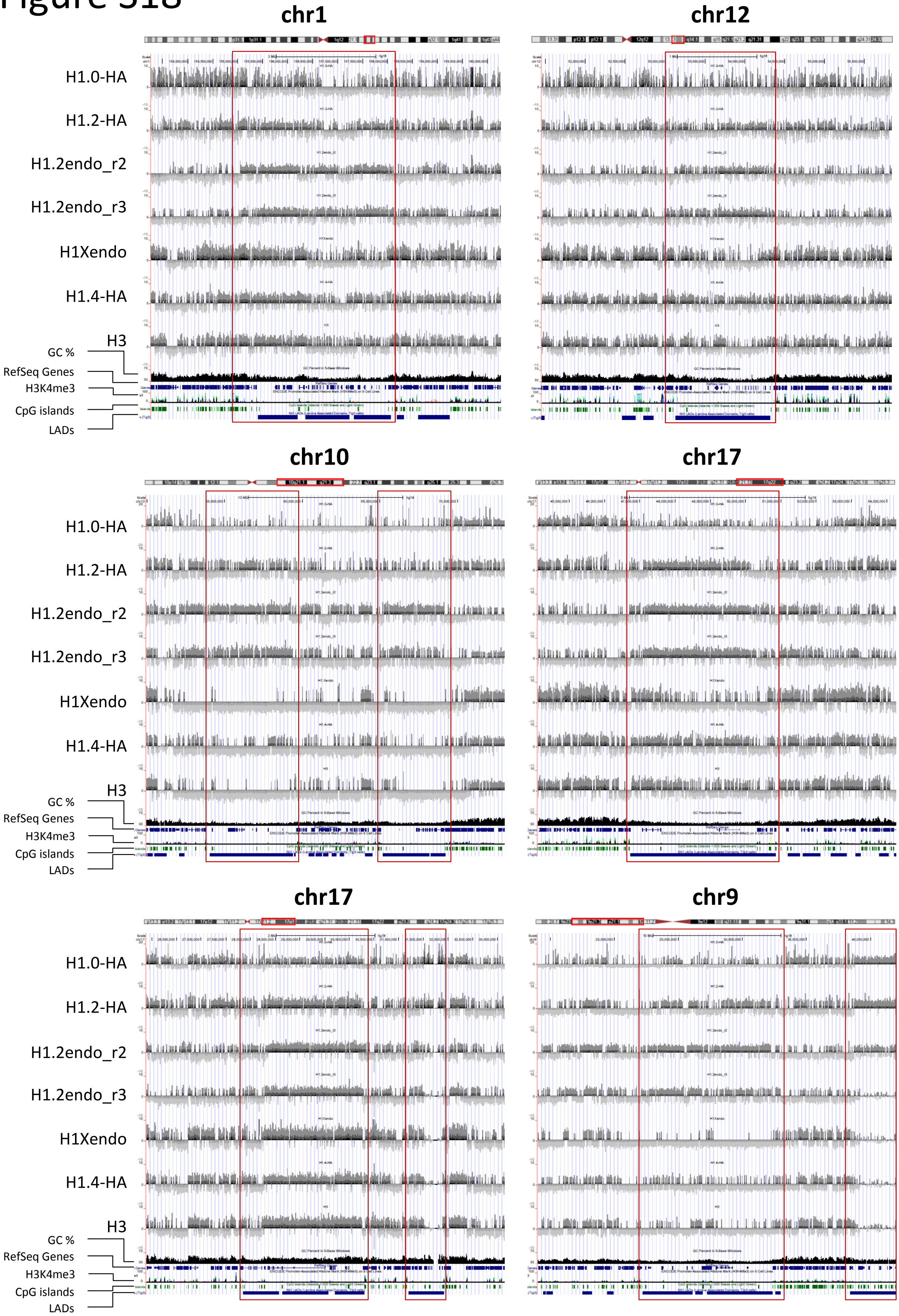


Millán-Ariño, Ll., et al.

# Figure S19

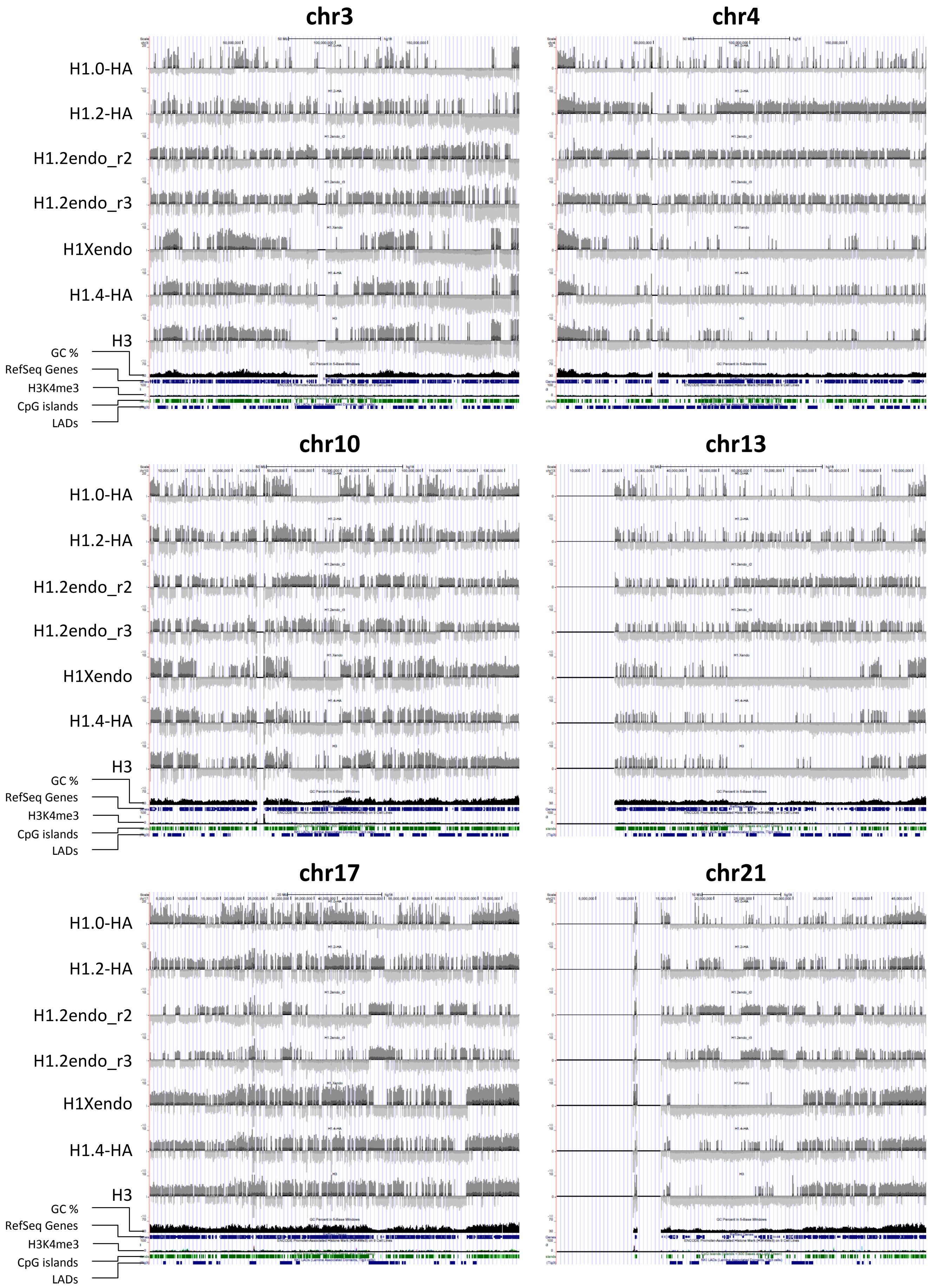


Millán-Ariño, Ll., et al.

# Figure S20

A

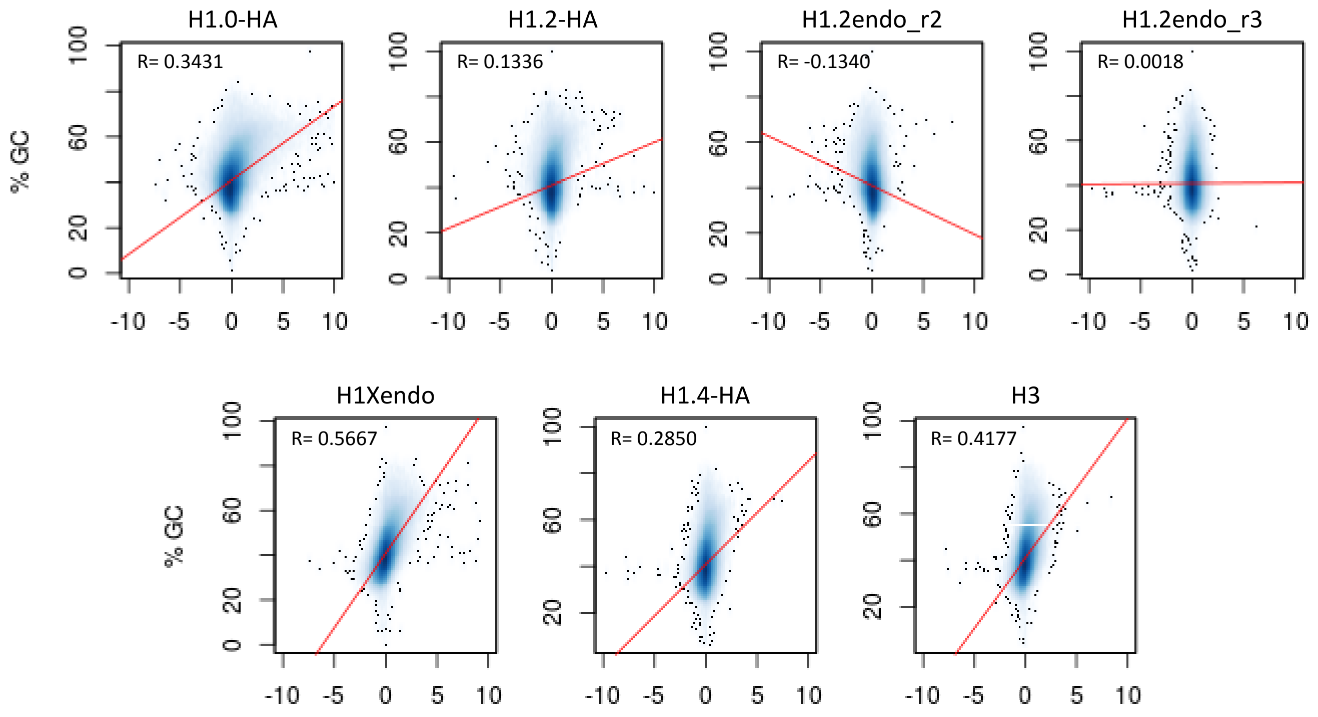


B

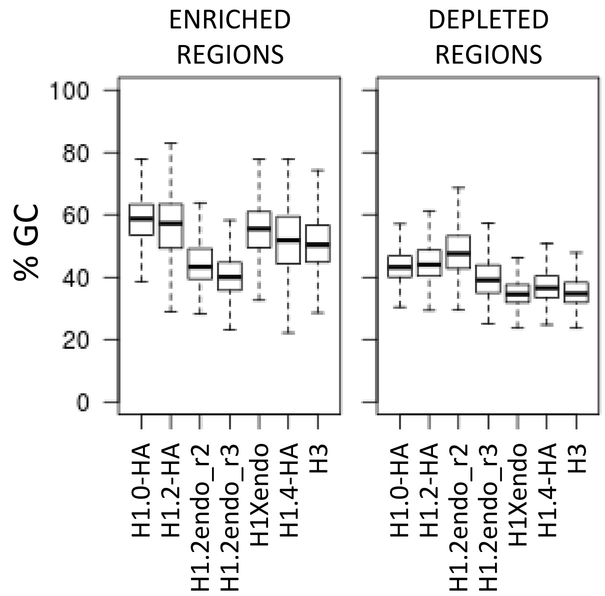


Millán-Ariño, Ll., et al.

# Figure S21

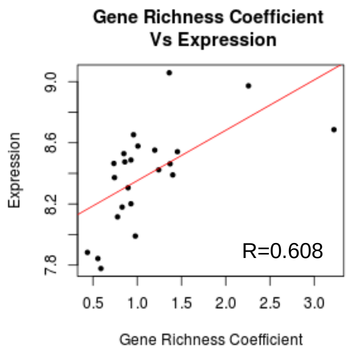


## Gene richness vs H1 abundance

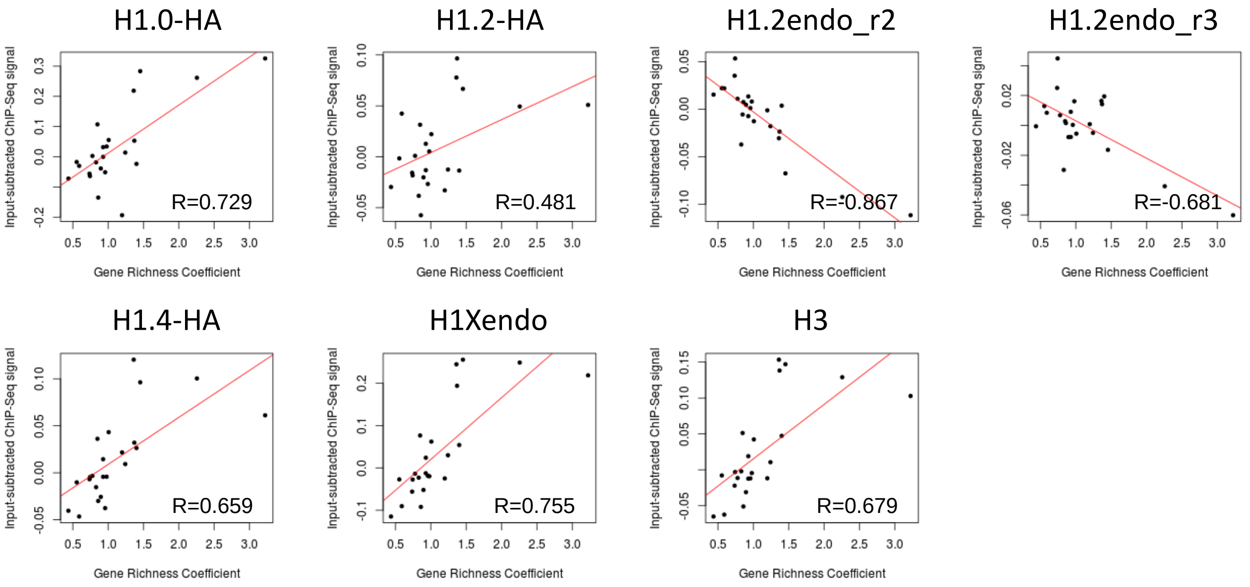


## Gene expression vs H1 abundance

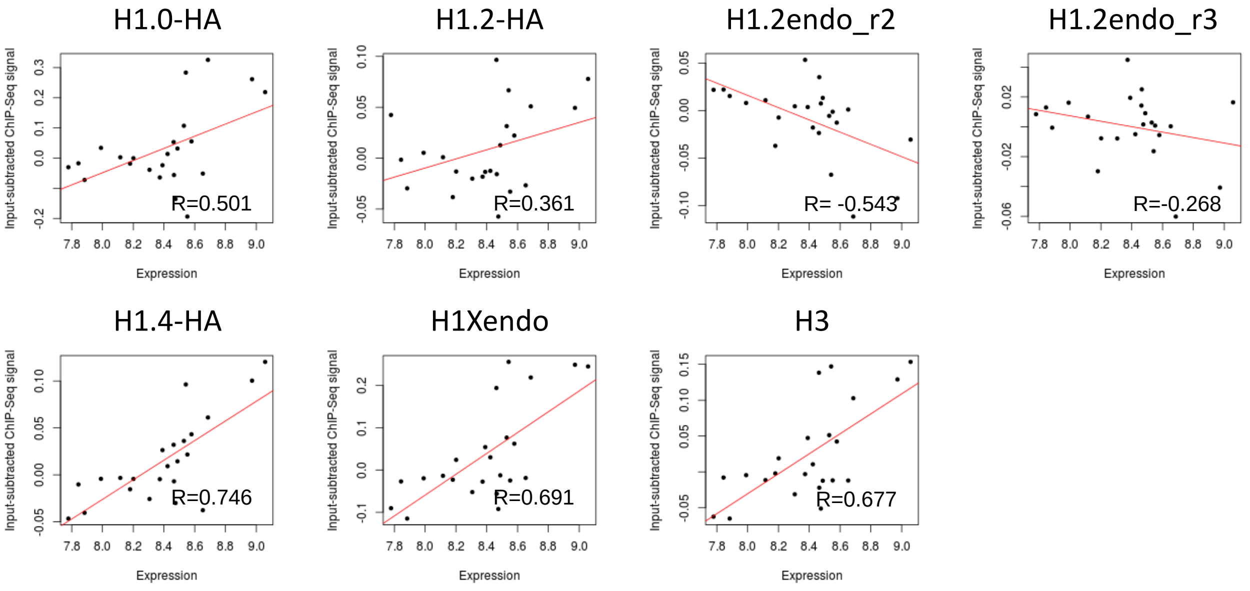

# Figure S22

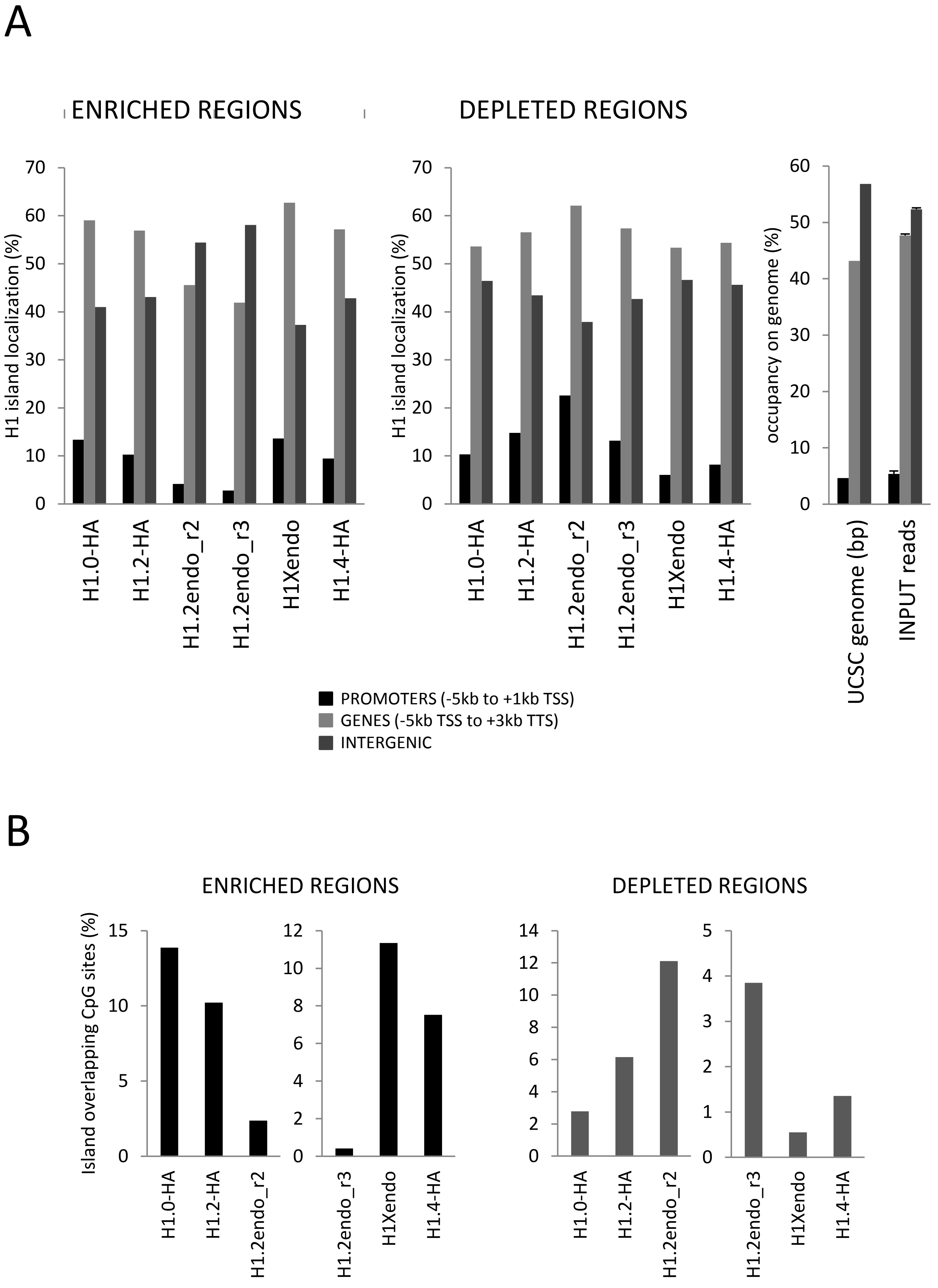


Millán-Ariño, Ll., et al.

# Figure S23

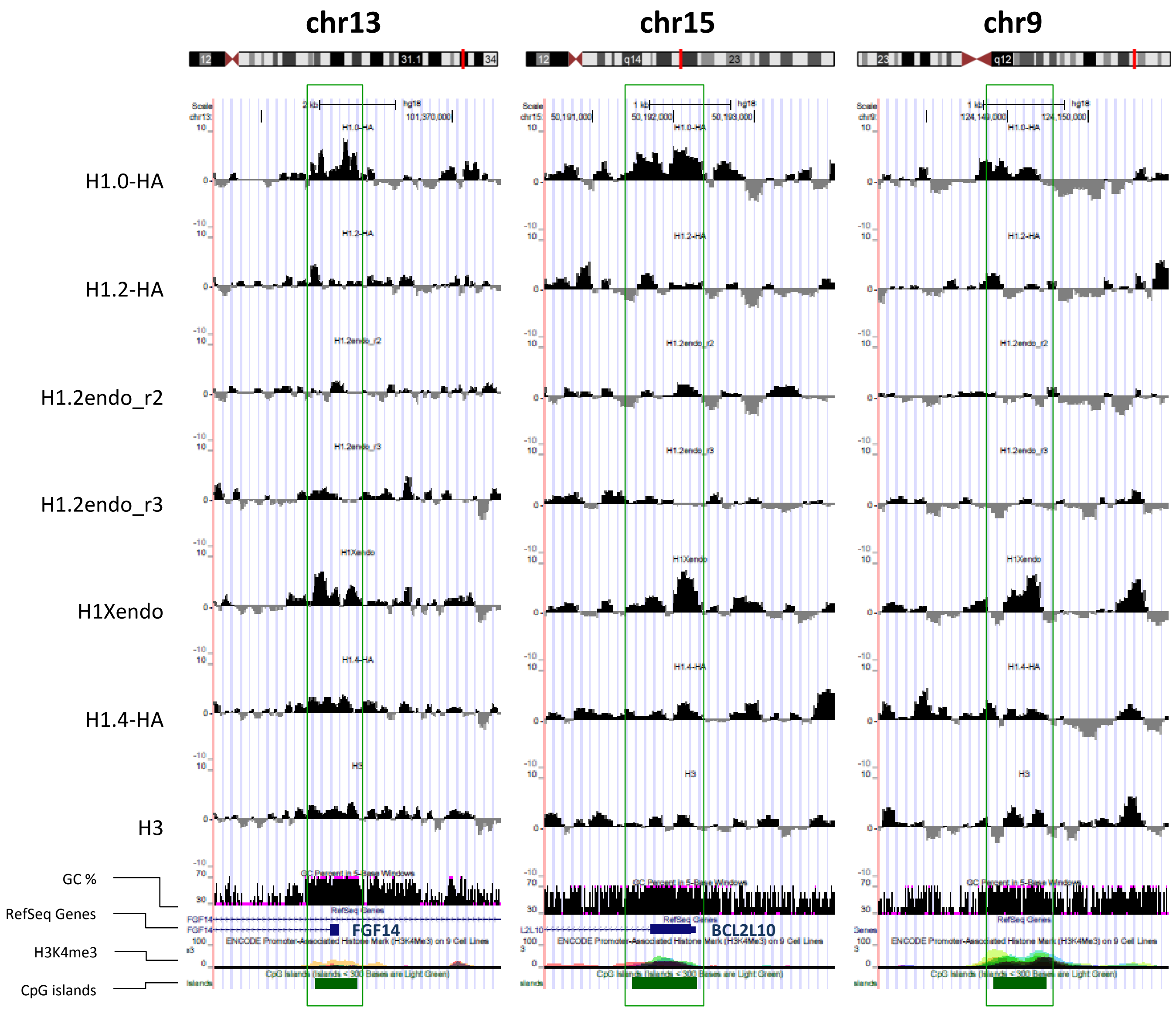


Millán-Ariño, Ll., et al.

# Figure S24

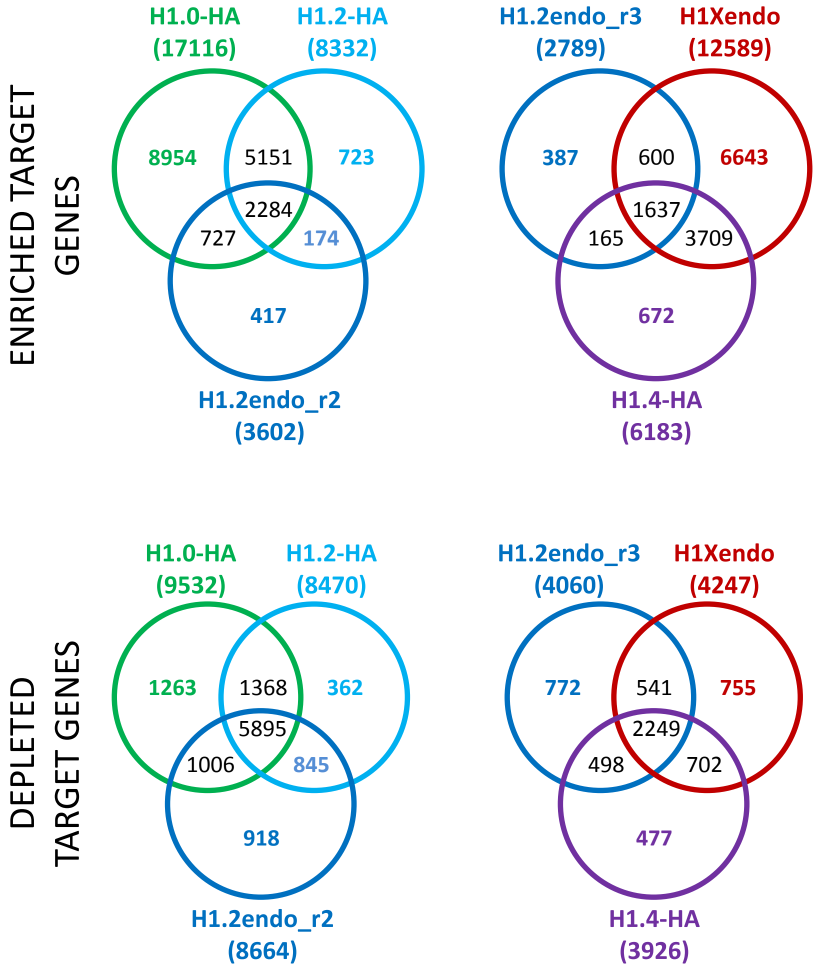
ENRICHED TARGET GENES
H1.0-HA
(17116)
H1.2-HA
(8332)
8954
5151
723
2284
727
174
417
H1.2endo_r2
(3602)
H1.2endo_r3
(2789)
H1Xendo
(12589)
387
600
6643
1637
165
3709
672
H1.4-HA
(6183)
DEPLETED TARGET GENES
H1.0-HA
(9532)
H1.2-HA
(8470)
1263
1368
362
5895
1006
845
918
H1.2endo_r2
(8664)
H1.2endo_r3
(4060)
H1Xendo
(4247)
772
541
755
2249
498
702
477
H1.4-HA
(3926)

# Figure S25

## ENRICHED REGIONS

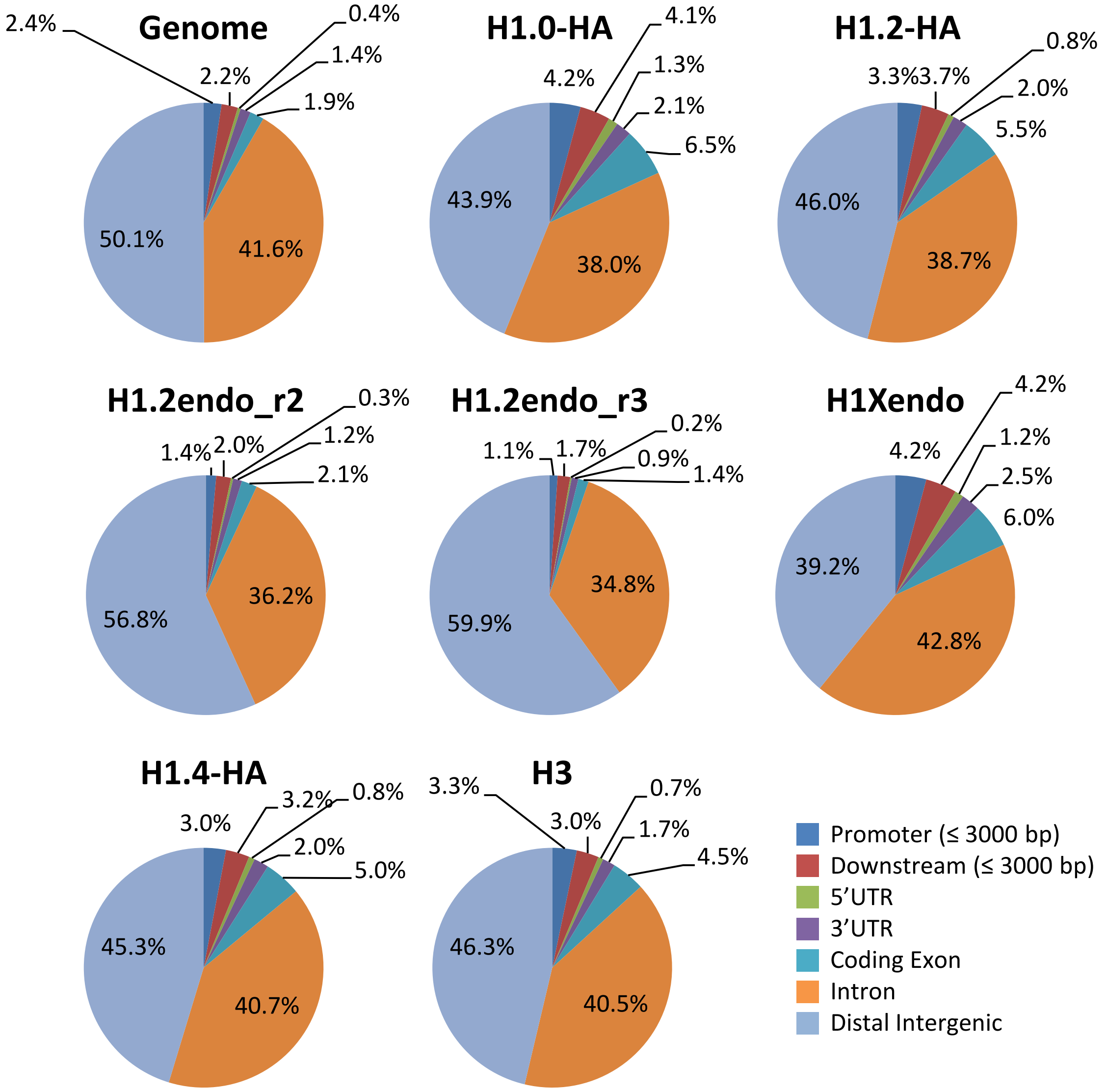


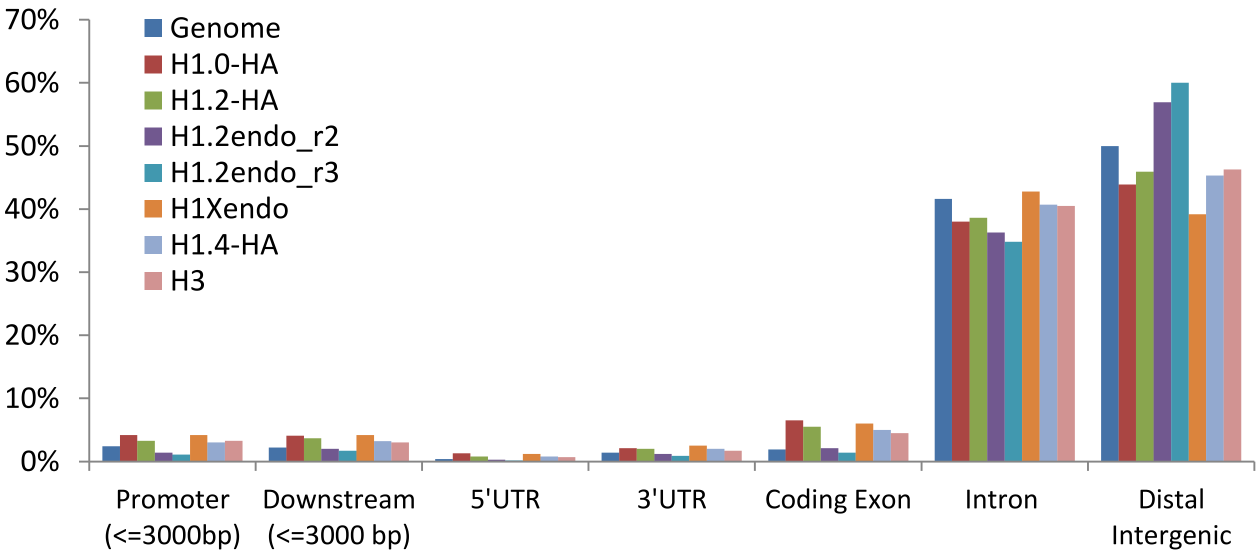


Millán-Ariño, Ll., et al.

# Figure S25

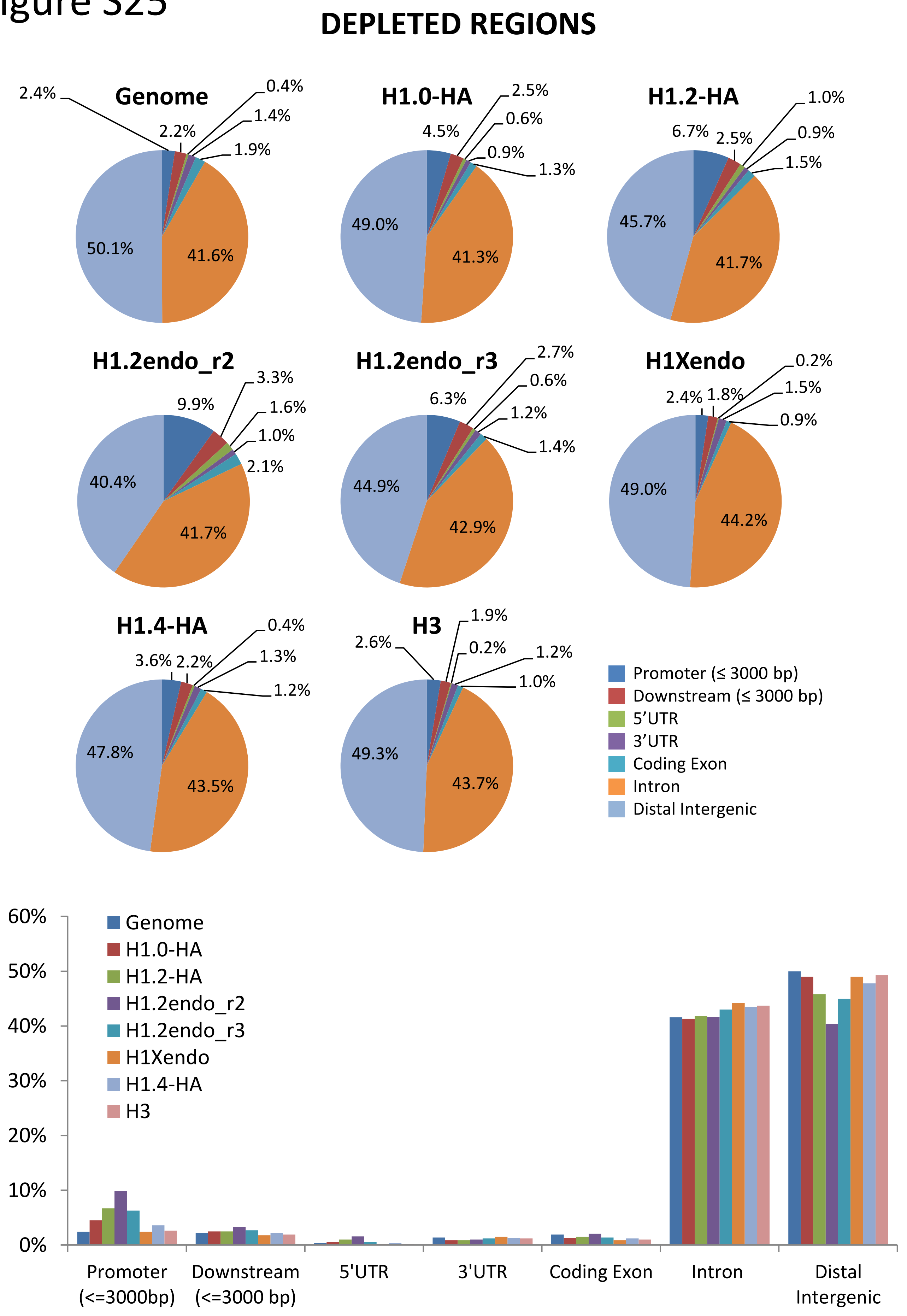


Millán-Ariño, Ll., et al.

# Table S1

List of read lenght, counts, and total mappable reads (to hg18) of the libraries

| Library | Read lenght (bp) | Total reads | Mapped reads | Mapped rate (%) |
|---|---|---|---|---|
| **H1.0-HA_r1** | 49 | 50,088,474 | 47,468,471 | 94.77 |
| **H1.2-HA_r1** | 49 | 49,572,689 | 46,963,903 | 94.74 |
| **H1.0-HA_r2** | 49 | 49,298,091 | 47,071,182 | 95.48 |
| **H1.2-HA_r2** | 49 | 50,163,521 | 48,076,368 | 95.84 |
| **H1.2endo_r2** | 49 | 50,380,892 | 48,487,915 | 96.24 |
| **INPUT_r2** | 49 | 51,302,633 | 48,832,661 | 95.19 |
| **H1.2endo_r3** | 49 | 51,112,949 | 49,120,181 | 96.10 |
| **H1Xendo_r3** | 49 | 51,304,089 | 49,152,136 | 95.81 |
| **H1.4-HA_r3** | 49 | 51,537,524 | 49,354,356 | 95.76 |
| **H3_r3** | 49 | 50,125,868 | 48,383,270 | 96.52 |
| **INPUT_r3** | 49 | 52,386,794 | 49,803,697 | 95.07 |

# Table S2

Gene ontology of H1.2endo top10% enriched promoters.

| **Biological Process** | **P-Value** | **Benjamini** |
|---|---|---|
| SENSORY PERCEPTION OF SMELL | 5.9E-45 | 2.1E-41 |
| SENSORY PERCEPTION OF CHEMICAL STIMULUS | 3.7E-43 | 6.4E-40 |
| SENSORY PERCEPTION | 9.0E-35 | 1.0E-31 |
| NEUROLOGICAL SYSTEM PROCESS | 4.9E-34 | 4.3E-31 |
| COGNITION | 1.2E-33 | 8.5E-31 |
| G-PROTEIN COUPLED RECEPTOR PROTEIN SIGNALLING PATHWAY | 3.9E-28 | 2.3E-25 |
| CELL SURFFACE RECEPTOR LINKED SIGNAL TRANSDUCTION | 1.9E-23 | 9.3E-21 |
| CELL ADHESION | 2.1E-5 | 9.1E-3 |
| BIOLOGICAL ADHESION | 2.2E-5 | 8.6E-3 |
| **Cellular Component** | **P-Value** | **Benjamini** |
| PLASMA MEMBRANE | 7.6E-11 | 3.9E-8 |
| INTEGRAL TO PLASMA MEMBRANE | 3.7E-10 | 9.5E-8 |
| INTRINSIC TO PLASMA MEMBRANE | 1.9E-9 | 3.3E-7 |
| EXTRACELLULAR REGION | 1.0E-5 | 1.4E-3 |
| EXTRACELLULAR REGION PART | 6.3E-5 | 6.5E-3 |

Gene ontology of H1.Xendo top10% enriched promoters.

| **Biological Process** | **P-Value** | **Benjamini** |
|---|---|---|
| NEURON DIFFERENTIATION | 5.6E-9 | 2.0E-5 |
| G-PROTEIN SIGNALLING, COUPLET TO CYCLIC NUCLEOTIDE SECOND MESSENGER | 6.8E-7 | 1.2E-3 |
| CELL-CELL SIGNALING | 1.1E-6 | 1.3E-3 |
| SECOND-MESSENGER-MEDIATED SIGNALLING | 1.4E-6 | 1.3E-3 |
| ANTERIOR/POSTERIOR PATTERN FORMATION | 1.9E-6 | 1.4E-3 |
| NEURON DEVELOPMENT | 2.9E-6 | 1.8E-3 |
| CYCLIC-NUCLEOTIDE-MEDIATED SIGNALLING | 3.2E-6 | 1.7E-3 |
| EMBRYONIC ORGAN DEVELOPMENT | 4.1E-6 | 1.9E-3 |
| PATTERN SPECIFICATION PROCESS | 4.5E-6 | 1.8E-3 |
| **Cellular Component** | **P-Value** | **Benjamini** |
| INTRINSIC TO PLASMA MEMBRANE | 1.0E-8 | 5.4E-6 |
| PLASMA MEMBRANE PART | 1.5E-8 | 3.9E-6 |
| PLASMA MEMBRANE | 2.8E-8 | 4.9E-6 |
| INTEGRAL TO PLASMA MEMBRANE | 4.5E-8 | 6.0E-6 |
| EXTRACELLULAR REGION | 2.5E-7 | 2.6E-5 |

Gene ontology of H1.2endo bottom10% enriched promoters.

| **Biological Process** | **P-Value** | **Benjamini** |
|---|---|---|
| REGULATION OF SYSTEM PROCESS | 1.1E-5 | 4.2E-2 |
| CELL-CELL SIGNALLING | 1.2E-5 | 2.4E-2 |
| SENSORY ORGAN DEVELOPMENT | 6.9E-5 | 8.6E-2 |
| NEGATIVE REGULATION OF CATABOLIC PROCESS | 1.1E-4 | 1.1E-1 |
| REGIONALIZATION | 2.7E-4 | 1.9E-1 |
| CIRCULATORY SYSTEM PROCESS | 8.5E-4 | 4.2E-1 |
| BLOOD CIRCULATION | 8.5E-4 | 4.2E-1 |
| **Cellular Component** | **P-Value** | **Benjamini** |
| PLASMA MEMBRANE PART | 1.1E-3 | 4.6E-1 |
| TROPONIN COMPLEX | 4.3E-3 | 7.1E-1 |
| DNA-DIRECTED RNA POLYMERASE II, CORE COMPLEX | 7.7E-3 | 7.7E-1 |
| PLASMA MEMBRANE | 1.5E-2 | 8.8E-1 |
| ACTIN CYTOSKELETON | 1.6E-2 | 8.4E-1 |

Gene ontology of H1Xendo bottom10% enriched promoters.

| **Biological Process** | **P-Value** | **Benjamini** |
|---|---|---|
| CHROMOSOME ORGANIZATION | 1.0E-11 | 3.4E-8 |
| CHROMATIN ORGANIZATION | 8.7E-11 | 1.4E-7 |
| NUCLEOSOME ORGANIZATION | 3.2E-6 | 3.5E-3 |
| CHROMTAIN MODIFICATION | 6.9E-6 | 5.6E-3 |
| NUCLEOSOME ASSEMBLY | 2.2E-5 | 1.4E-2 |
| DNA PACKING | 2.4E-5 | 1.3E-2 |
| PROTEIN-DNA COMPLEX ASSEMBLY | 2.5E-5 | 1.2E-2 |
| **Cellular Component** | **P-Value** | **Benjamini** |
| INTRACELLULAR ORGANELLE LUMEN | 7.2E-7 | 4.2E-4 |
| ORGANELLE LUMEN | 1.2E-6 | 3.4E-4 |
| MEMBRANE-ENCLOSED LUMEN | 2.5E-6 | 4.8E-4 |
| NUCLEAR LUMEN | 8.8E-6 | 1.3E-3 |
| CHROMOSOME | 1.3E-5 | 1.5E-3 |

# Table S3

| ENRICHED ISLANDS | H1.0-HA | H1.2-HA | H1.2endo_r2 | H1.2endo_r3 | H1Xendo | H1.4-HA |
|---|---|---|---|---|---|---|
| Total islands | 49320 | 16059 | 7500 | 6911 | 38782 | 12478 |
| % intergenic/total | 40.97 | 43.10 | 54.44 | 58.10 | 37.29 | 42.84 |
| % genic/total | 59.03 | 56.90 | 45.56 | 41.90 | 62.71 | 57.16 |
| % promoter/gene | 22.68 | 18.01 | 9.13 | 6.63 | 21.75 | 16.49 |
| Target genes | 17116 | 8332 | 3602 | 2789 | 12589 | 6183 |
| Target promoters | 7591 | 2176 | 490 | 257 | 6284 | 1715 |
| % CpG overlap | 13.88 | 10.22 | 2.37 | 0.42 | 11.35 | 7.53 |

| DEPLETED ISLANDS | H1.0-HA | H1.2-HA | H1.2endo_r2 | H1.2endo_r3 | H1Xendo | H1.4-HA |
|---|---|---|---|---|---|---|
| Total islands | 25459 | 15224 | 12305 | 5714 | 10205 | 6864 |
| % intergenic/total | 46.41 | 43.44 | 37.89 | 42.65 | 46.65 | 45.63 |
| % genic/total | 53.59 | 56.56 | 62.11 | 57.35 | 53.35 | 54.37 |
| % promoter/gene | 19.28 | 26.13 | 36.35 | 23.02 | 11.34 | 15.06 |
| Target genes | 9532 | 8470 | 8664 | 4060 | 4247 | 3926 |
| Target promoters | 4105 | 3783 | 4610 | 1412 | 1014 | 1046 |
| % CpG overlap | 2.78 | 6.15 | 12.11 | 3.85 | 0.55 | 1.35 |

**Supplementary Methods**

**Treatments**

For H1 knock-down cell lines, doxycycline (Sigma) was added at 2.5 $\mu$g/ml when indicated. Along a 6-day treatment with Dox, cells were passaged at day 3.

For hormone treatment experiments with R5020 (PerkinElmer Life Sciences), cells were plated in phenol red-free medium supplemented with 10% dextran-coated charcoal-treated FBS and, 24 hours later, the medium was replaced by fresh serum-free medium. After 24 hours under serum-free conditions, cells were treated with R5020 (10 nM) for different times at 37ºC.

**Antibodies**

Polyclonal antibodies specifically recognizing human H1 variants, are: anti-H1.0 (Abcam 11079), anti-H1 phospho-T146 (Abcam 3596), anti-H1.5 (Abcam 18208). Other antibodies used are: anti-H3K4me3 (Abcam 8580), and anti-total H1 (Millipore, clone AE-4, 05-457).

**H1 Extraction, Gel Electrophoresis and Immunoblotting**

Histone H1 was purified by 5% perchloric acid lysis for 1 hour at 4ºC. Soluble acid proteins were precipitated with 30% trichloroacetic acid over-night at 4ºC, washed twice with 0.5 ml of acetone and reconstituted in water. Protein concentration was determined by Micro BCA protein assay (Pierce).

Chromatin or purified H1 histones were subjected to 12% SDS-PAGE, transferred to a PVDF membrane, blocked with Odissey blocking buffer (LI-COR Biosciences) for 1 hour, incubated with primary antibodies over-night at 4ºC and with secondary antibodies conjugated to fluorescence (IRDye 680 goat anti-rabbit IgG and IRDye 800CW goat anti-mouse IgG, LI-COR) for 1 hour at room temperature. Bands were visualized with the Odissey Infrared Imaging System.

**Formaldehyde-Assisted Isolation of Regulatory Elements (FAIRE) Assays**

Cells were fixed using 1% formaldehyde, harvested and sonicated using a Diagenode Bioruptor to generate chromatin fragments between 200 and 500 bp. To prepare input DNA an aliquot of chromatin was taken treated with RNAase A, de-crosslinked overnight at 65ºC, purified by phenol/chloroform extraction, and run on a gel to ensure

average fragment sizes of 200-500 bp. FAIRE DNA was prepared processing twice chromatin by phenol/chloroform extraction to purify DNA not bound by nucleosome in the water phase. The samples were later treated with RNase A, de-crosslinked by overnight incubation at 65ºC, and purified by GenElute PCR Clean-Up Kit (Sigma). Real-time PCR was performed on FAIRE and input DNA using EXPRESS SYBR GreenER qPCR SuperMix Universal from Invitrogen and specific oligonucleotides in a Roche 480 Lightcycler. All oligonucleotide sequences used for the amplifications are available on request.

**RNA extraction and RT-PCR**

Total RNA was extracted using High Pure RNA isolation kit (Roche) according to the manufacturer's instructions. cDNA was obtained from 100 ng of total RNA using SuperScript VILO cDNA synthesis (Invitrogen). Gene products were analyzed by qPCR using EXPRESS SYBR GreenER qPCR SuperMix Universal (Invitrogen) and specific oligonucleotides in a Roche 480 Lightcycler. Each value was corrected by human GAPDH and expressed as relative units. Gene-specific oligonucleotide sequences are available on request.
